# Integrated control of *Aedes albopictus* in Southwest Germany supported by the Sterile Insect Technique

**DOI:** 10.1186/s13071-021-05112-7

**Published:** 2022-01-05

**Authors:** Norbert Becker, Sophie Min Langentepe-Kong, Artin Tokatlian Rodriguez, Thin Thin Oo, Dirk Reichle, Renke Lühken, Jonas Schmidt-Chanasit, Peter Lüthy, Arianna Puggioli, Romeo Bellini

**Affiliations:** 1 Faculty of Biosciences, University of Heidelberg, Im Neuenheimer Feld 230, 69120 Heidelberg, Germany; 2 Institute of Dipterology (IfD), Georg-Peter-Süß-Str. 3, 67346 Speyer, Germany; 3 Kommunale Aktionsgemeinschaft zur Bekämpfung der Schnakenplage e.V. (KABS), Georg-Peter-Süß-Str. 3, 67346 Speyer, Germany; 4 IcyBac–Biologische Stechmückenbekämpfung GmbH (ICYBAC), Georg-Peter-Süß-Str. 1, 67346 Speyer, Germany; 5 Department of Arbovirology, Bernhard-Nocht-Institute for Tropical Medicine, Bernhard-Nocht-Str. 74, 20359 Hamburg, Germany; 6 Faculty of Mathematics, Informatics and Natural Sciences, Universität Hamburg, Ohnhorststrasse 18, 22609 Hamburg, Germany; 7 Institute of Microbiology, Swiss Federal Institute of Technology (ETH Zürich), Vladimir-Prelog-Weg 1-5/10, 8093 Zürich, Switzerland; 8 Centro Agricoltura Ambiente “G. Nicoli” (CAA), Via Sant’Agata 835, 40014 Crevalcore, Italy

**Keywords:** *Aedes albopictus*, Distribution, Integrated control, *Bacillus thuringiensis israelensis*, Sterile insect technique, Germany

## Abstract

**Background:**

The invasive species *Aedes albopictus*, commonly known as the Asian tiger mosquito, has undergone extreme range expansion by means of steady introductions as blind passengers in vehicles traveling from the Mediterranean to south-west Germany. The more than 25 established populations in the State of Baden-Württemberg, Palatine and Hesse (south-west Germany) have become a major nuisance and public health threat. *Aedes albopictus* deserves special attention as a vector of arboviruses, including dengue, chikungunya and Zika viruses. In Germany, *Ae. albopictus* control programs are implemented by local communities under the auspices of health departments and regulatory offices.

**Methods:**

The control strategy comprised three pillars: (i) community participation (CP) based on the elimination of breeding sites or improved environmental sanitation, using fizzy tablets based on* Bacillus thuringiensis israelensis* (fizzy Bti tablets; Culinex® Tab plus); (ii) door-to-door (DtD) control by trained staff through the application of high doses of a water-dispersible Bti granular formulation (Vectobac® WG) aimed at achieving a long-lasting killing effect; and (iii) implementation of the sterile insect technique (SIT) to eliminate remaining *Ae. albopictus* populations. Prior to initiating large-scale city-wide treatments on a routine basis, the efficacy of the three elements was evaluated in laboratory and semi-field trials. Special emphasis was given to the mass release of sterile *Ae. albopictus* males.

**Results:**

More than 60% of the local residents actively participated in the first pillar (CP) of the large-scale control program. The most effective element of the program was found to be the DtD intervention, including the application of Vectobac® WG (3000 ITU/mg) to potential breeding sites (10 g per rainwater container, maximum of 200 l = maximum of approx. 150,000 ITU/l, and 2.5 g per container < 50 l) with a persistence of at least 3 weeks. In Ludwigshafen, larval source management resulted in a Container Index for *Ae. albopictus* of < 1% in 2020 compared to 10.9% in 2019. The mean number of *Aedes* eggs per ovitrap per 2 weeks was 4.4 in Ludwigshafen, 18.2 in Metzgergrün (Freiburg) (SIT area) and 22.4 in the control area in Gartenstadt (Freiburg). The strong reduction of the *Ae. albopictus* population by Bti application was followed by weekly releases of 1013 (Ludwigshafen) and 2320 (Freiburg) sterile *Ae. albopictus* males per hectare from May until October, resulting in a high percentage of sterile eggs. In the trial areas of Ludwigshafen and Frieburg, egg sterility reached 84.7 ± 12.5% and 62.7 ± 25.8%, respectively; in comparison, the natural sterility in the control area was 14.6 ± 7.3%. The field results were in line with data obtained in cage tests under laboratory conditions where sterility rates were 87.5 ± 9.2% after wild females mated with sterile males; in comparison, the sterility of eggs laid by females mated with unirradiated males was only 3.3 ± 2.8%. The overall egg sterility of about 84% in Ludwigshafen indicates that our goal to almost eradicate the *Ae. albopictus* population could be achieved. The time for inspection and treatment of a single property ranged from 19 to 26 min depending on the experience of the team and costs 6–8 euros per property.

**Conclusions:**

It is shown that an integrated control program based on a strict monitoring scheme can be most effective when it comprises three components, namely CP, DtD intervention that includes long-lasting Bti-larviciding to strongly reduce *Ae. albopictus* populations and SIT to reduce the remaining *Ae. albopictus* population to a minimum or even to eradicate it. The combined use of Bti and SIT is the most effective and selective tool against *Ae. albopictus*, one of the most dangerous mosquito vector species.

**Graphical Abstract:**

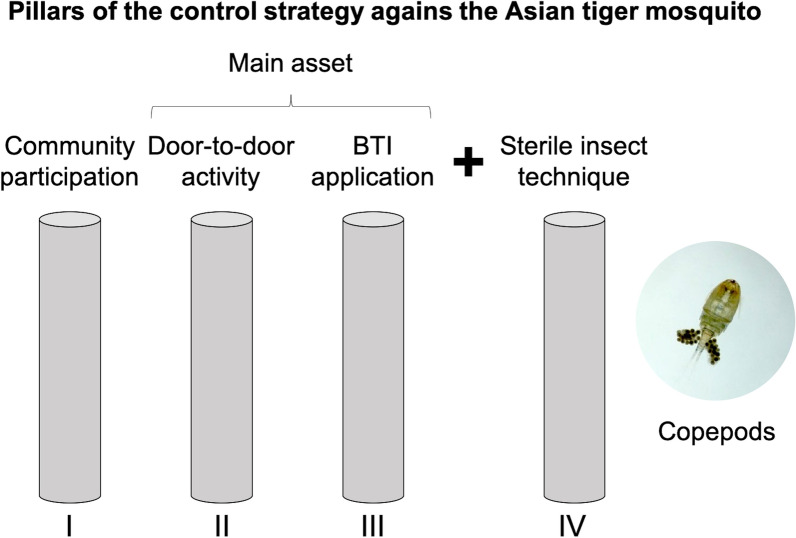

**Supplementary Information:**

The online version contains supplementary material available at 10.1186/s13071-021-05112-7.

## Background

Among the more than 3500 known mosquito species, about 30 have spread beyond their original geographical borders [1–3]. Several invasive species have a severe impact on public health, not only in the tropics but also in temperate climates, including *Aedes aegypti*, *Ae. albopictus*, *Ae. japonicus*, *Ae. koreicus*, *Ae. atropalpus* and *Ae. triseriatus*.

*Aedes albopictus* deserves special attention as a vector of at least 22 arboviruses, including dengue, chikungunya, Zika and yellow fever viruses [4–8]. Since 1990, *Ae. albopictus* has had a permanent foothold in Europe, likely first introduced to Italy in used tires as mode of invasion via the international trade in used tires. From Genoa [9, 10], *Ae. albopictus* spread as a “blind” passenger via road, rail and boat transport systems across Italy and further along the Mediterranean coast to France, Spain, the Balkans, Greece and Turkey [11]. Today, the species is firmly established in the whole European Mediterranean basin and has started spreading across the Alps into central Europe. Autochthonous transmissions of chikungunya, dengue and Zika viruses vectored by *Ae. albopictus* have flared up in the Mediterranean region [6–8, 12–15].

**Fig. 1:**
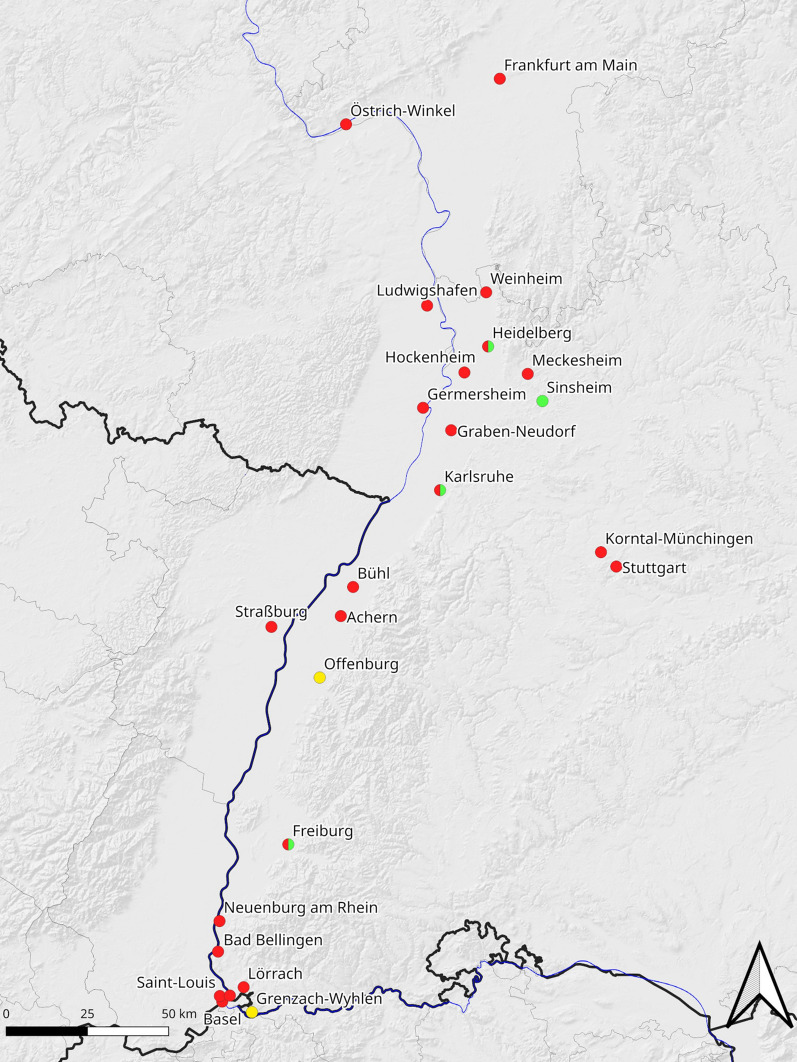
*Aedes albopictus* populations recorded in 2019. Color coding: Red, not controlled so far; green, eradicated; green–red, strongly reduced; yellow, population size not known

Realizing the risk of passive transport of *Ae. albopictus* from Italy to Germany by road and rail, especially during the summer holiday season, the German Mosquito Control Association (KABS) started a monitoring program in 2005 [2]. Surveillance of rest areas and camping grounds along the A5 motorway leading from Italy revealed a regular and increasing appearance of *Ae. albopictus* along the upper Rhine area during the summer months [2, 16, 17]. The first established population was reported in an allotment garden in Freiburg in September 2014 [18]. By 2019 at least 20 cities along the upper Rhine valley were infested by overwintering populations of *Ae. albopictu*s (Fig. 1).

In all colonized areas, immediate surveillance was initiated by means of ovitraps, larval sampling and human bait collections to assess the size of the infested area and the abundance of *Ae. albopictus*. The goal was to detect and eliminate the breeding sites and to start eradication programs. After an in-depth evaluation of control tools in a previous study [19], the decision was made to base the control strategy on three pillars: (i) community participation (CP), including distribution of fizzy tablets containing the protoxins of *Bacillus thuringiensis israelensis* (Bti); (ii) door-to-door (DtD) activities, including the elimination/removal or treatment of all breeding sites by trained staff using a Bti suspension at about 4-week intervals; and (iii) the integration of the sterile insect technique (SIT), an environment-friendly insect pest control method, to wipe out remaining *Ae. albopictus* populations originated from cryptic and/or non-accessible breeding sites.

The CP approach focused on increasing the public awareness to prevent mosquito breeding and to record the occurrence of *Ae. albopictus* as an “early warning system.” It included providing detailed information to the public via press releases, TV air time, flyers, web pages and information events, such as at schools, in city halls or meetings of garden associations. Thus, public awareness was strengthened by providing detailed information on the characteristics, distribution and biology of the Asian tiger mosquito. The CP pillar of the program also involved the communication of measures to be undertaken to prevent the proliferation of the mosquito. These measures included the elimination of breeding sites and improved environmental sanitation, such as through the use of firmly fitting lids to water containers and the treatment of water with Bti fizzy tablets (Culinex® Tab plus; Culinex Becker GmbH, Ludwigshafen am Rhein, Germany). Bti fizzy tablets were distributed to the public in support of the DtD activities conducted by the expert teams. Additionally, in heavily infested areas, mosquito nets were distributed free of charge to encourage people to thoroughly cover rainwater containers, thereby preventing access to female mosquitoes looking for locations to oviposit.

However, due to the lack of professional know-how and active involvement, CP alone was not enough to reach the goal of strongly reducing or even eliminating the Asian tiger mosquito populations [20]. Therefore, a DtD program involving trained staff was implemented to control *Ae. albopictus* along with long-lasting Bti treatments.

The final goal was a significant reduction or—ideally—the elimination of *Ae. albopictus* populations. Therefore, SIT was added as third pillar to the integrated control strategy using gamma-irradiated sterile males. *Aedes albopictus* is particularly suitable for employing SIT, as the species is easily mass-reared, having a limited flight range, does not reproduce in enormous masses within a very short period like floodwater mosquitoes and breeding sites are well defined and mainly in urban areas [21]. Therefore, the SIT method was considered to be an excellent tool to access those breeding sites that were beyond the standard control measures, as well as properties whose owners refused entry permission.

Preceding the release of sterile males, the natural *Ae. albopictus* population has to be low or strongly reduced by earlier CP and DtD control initiatives. The sterile males have to outcompete their wild counterparts, and when successful, the result is a large majority of wild females laying sterile eggs [22].

The goal of the pilot program was to assess the effect of the three-pillar control strategy against *Ae. albopictus* starting in the laboratory, extending to semi-field tests and ending in routine field applications. Ovitraps served as the main monitoring tool. A cost analysis is presented to serve as a guideline for further planning of community-based control activities.

## Methods

### Study areas

The large-scale study was conducted in three large areas in southern Germany infested with *Ae. albopictus*.

#### The Melm district (65 ha) within the city limits of Ludwigshafen (Palatine)

**Fig. 2:**
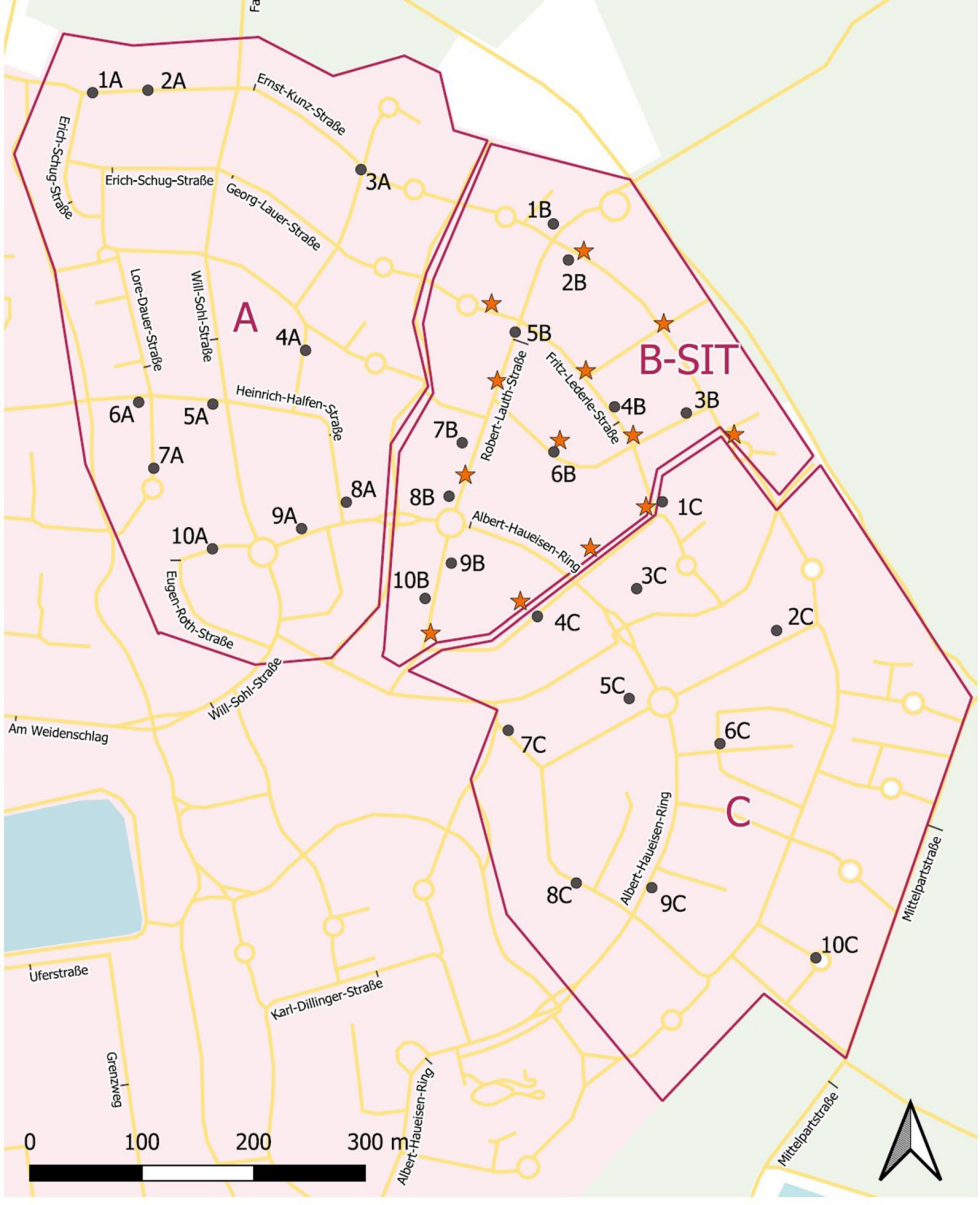
Map of the Melm study area, city of Ludwigshafen, showing the sectors (A, B, C), positions of ovitraps (black dots & identification number of trap) and release spots for sterile males (stars). Abbreviations: SIT, Sterile insect technique. Contributors: GeoBasis-DE/LVermGeoRP2020, 2018, Geofabrik GmbH, Karlsruhe, Germany/OpenStreetMap)

The Melm district is a well-defined residential area, originally developed at the end of the last century. It consists of about 1000 properties with gardens and some apartment buildings. Abundant breeding sites of *Ae. albopictus* were present. The 65-ha study site was subdivided into three sectors of almost equal size, each with specific mosquito control scheme: sector A, 23 ha (CP + DtD); sector B, 17 ha (CP + DtD + SIT); sector C, 25 ha (CP + DtD) (Fig. 2).

#### The Metzgergrün area within the city of Freiburg (Baden-Württemberg)

**Fig. 3:**
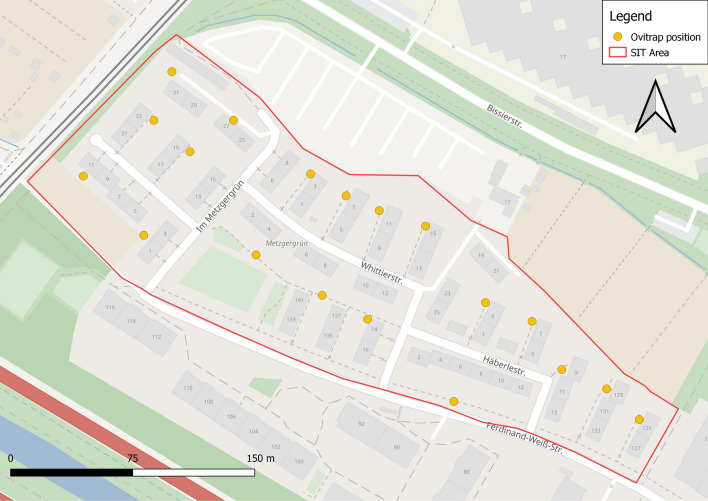
Map of the Metzgergrün study area, city of Freiburg, where SIT, in addition to CP and DtD, was applied overall. Area outlined in red indicates the release area; solid yellow circles indicate the location of ovitraps. Abbreviations: CP, Community participation activities; DtD, door-to-door activities

The Metzgergrün area has a size of 4.5 ha comprising mostly apartment buildings for social housing (Fig. 3). A large number of potential breeding sites, such as used tires and water-catching garbage, are present in adjacent garden sites. In this area, SIT was employed in addition to CP and DtD.

#### Gartenstadt (Freiburg)

The Gartenstadt site served as control area (4 ha) and is located about 1 km away from Metzgergrün. The infestation by *Ae. albopictus* at this site was comparable to that at Metzgergrün.

### Design and layout

The large-scale field study was based on a three-pillar strategy, namely CP, DtD and SIT.

#### Community participation

In the first week of May 2020, prior to the start of the program, citizens in Melm were informed via the local media about the planned control activities against the Asian tiger mosquito. The goal was to turn the residents from spectators to actors in the fight against *Ae. albopictus*. On 11 and 12 May, two persons distributed flyers to 1820 households with detailed information that would help residents control the Asian tiger mosquito population(s) on their properties. Specifically, the flyer delivered to each household contained instructions for control and  how to use the 10 fizzy Bti tablets in a blister pack (Culinex® Tab plus, lot: 0604783, activity: 1000 international toxic units [ITU]/mg) contained in the cardboard box attached to the flyer.

The DtD activities included recording the number of accessed and non-accessed properties as well as the presence of breeding sites or container-breeding mosquitoes, with the aim to determine the efficacy of CP. Furthermore, the residents were interviewed regarding their knowledge of mosquito control. During the last control activity (round no. 5) in September, all available residents were asked if they had implemented the proposed control activities on their property.

#### DtD control

We considered the DtD approach in which trained staff applied long-lasting Bti treatments to be the most powerful tool to control *Ae. albopictus *[23]. Therefore, our critical first step was to determine the optimum dosage for the Bti treatments in large and small water collection vessels. All Bti formulations are sterilized by gamma radiation at a dose of 25 kGy [24].

##### Assessment of the optimum effective dosage for Bti treatments

Whereas the control of floodwater mosquitoes requires only a single effective dosage of Vectobac® WG, a water-dispersible granular formulation of Bti (strain AM5265; Valent Biosciences, Libertyville, IL, USA) [25], the control of container-breeding species needs a product with long-term residual activity due to the constant follow-up of generations. Thus, both the number of retreatments and the manpower costs could be strongly reduced when a long-term effect of several weeks (at least 3 weeks) is achieved.

In a first series of tests, the effect of Bti treatments in rainwater containers was simulated. The Bti test mixture (stock solution) was prepared by thorougly mixing 250 g of Vectobac® WG (activity: 3000 ITU/mg) with 1.5 l of tap water (pH: 7.8; conductivity: 680 µS/cm; 0.75 × 10^9^ ITU per 1500 ml). Aliquots of 6, 0.6 and 0.06 ml, respectively, were removed from this stock suspension, under conditions of constant stirring, by Eppendorf pipette and added to plastic buckets containing 20 l of tap water (1/10 of the usual water volume of a regular rainwater container), corresponding to 1, 0.1 and 0.01 g of Vectobac® WG per bucket, respectively. Each dosage was tested in four replicates at a constant temperature of 24 °C ± 1 °C, with four buckets serving as a control. Twenty third-instar larvae of laboratory-reared *Ae. albopictus* were added to each bucket, and mortality was recorded at 24 and 48 h post-treatment. At weekly intervals, 5 l of water was removed and replaced with the same amount of tap water to simulate natural conditions when water is removed for watering the garden. Then, 20 third-instar larvae were again added to each container. At each 48-h reading, larval cadavers were removed. The experiment was run until the mortality rates fell to < 60%.

In a second series of tests, the efficacy of Vectobac® WG was assessed in four different small water containers typically present in garden areas: (i) terracotta flowerpots with rough surfaces (volume: 1400 ml); (ii) terracotta pots with smooth walls (volume: 1400 ml); (iii) plastic flowerpots (volume: 950 ml); (iv) zinc pots (volume: 800 ml); and (v) in terracotta flower pot saucers (volume: 200 ml). Before the start of the treatment, the four flower pots were scrubbed with commercial potting soil (Compo Sana; Compo GmbH, Münster, Germany), cleaned with water and dried for 24 h to simulate natural conditions. The inside each type of empty container was homogenously sprayed with 15 ml of Vectobac® WG stock solution (as in the first series of tests: 250 g Vectobac® WG mixed with 1.5 l tap water; 0.75 × 10^9^ ITU/1500 ml) using a pressurized sprayer (Mesto Bugsi 1.5 L; MESTO Spritzenfabrik Ernst Stockburger GmbH, Freiberg am Neckar, Germany), resulting in 2.5 g Vectobac® WG per small container (7.5 × 10^6^ ITU per container). The containers were then dried for 48 h and filled with tap water. Twenty *Ae. albopictus* third-instar larvae were added to each container, and mortality was recorded at 48 h post-treatment. The containers were emptied after each mortality reading, dried again for 48 h, refilled with water and stocked with a new batch of larvae. The procedure was repeated until the mortality rates fell to < 60%. Four containers of each type served as untreated control. The test was conducted at 24 °C ± 1.5 °C and 80% relative humidity (RH).

##### Routine Bti treatments

The field staff consisted of six persons with in-depth knowledge of mosquito biology and taxonomy, especially the breeding habits of *Ae. albopictus*. The field staff was tasked with applying Bti at predetermined dosages to each remaining potential breeding site as well as evaluating the mosquito traps at regular intervals for surveillance purposes. All accessible properties were inspected from June to October at 4-week intervals. All remaining potential breeding sites were treated with Vectobac® WG. An essential role of the field staff was to keep close contact with the residents of > 1800 households, providing information and Bti-tablets as needed. Between May and September, the program included five rounds of surveillance, control and data collection. The data were entered into a geographic information system (GIS).

In Melm, biting *Ae. albopictus* were observed for the first time in early August 2019, and the county health department and city authorities were informed immediately. *Aedes albopictus* populations were found along four streets, and more breeding sites (unused flowerpots, saucers, rainwater barrels) were also identified scattered across the district. Gullies were not functioning as breeding sites because all the water collected in gullies runs off directly into the sewage plants. In total, 55 potential breeding sites were inspected, of which six tested positive for *Ae. albopictus* (Container Index [CI]: 10.9%). Thus, in addition to the identification of *Ae. albopictus* based on complaints of residents in 2019, the existence of a widespread reproducing population was documented. In the middle of August 2019, representatives of the health department, city authorities and specialists from KABS/Institute of Dipterology (IfD) met to discuss and coordinate further actions. In a first step, the residents were informed via media, and a website was created as a reporting platform. In the last week of August 2019, all households received flyers and Bti tablets for self-help. Several citizens reported severe nuisance caused by *Ae. albopictus* in their garden area. An action plan was designed together with authorities that served as a concept for an integrated control strategy in 2020. The plan included:

1. Press releases and information for the public through local media in close cooperation with the local authorities (first half of May 2020).
2. Training of field staff (6 people) in early May, which included information/training on: (i) the biology and taxonomy of mosquitoes in order to be able to distinguish between *Ae. albopictus* and other container-breeding mosquitoes, such as *Culex pipiens* sensu lato/*Cx. torrentium*, *Culiseta annulata*, *Culiseta longiareolata* or *Aedes japonicus*; (ii) the breeding habitats of *Ae. albopictus* and how to identify and enter the breeding sites in a database; (iii) the correct application of the biological larvicide Bti; (iv) the handling of mosquito traps; and (v) how to approach residents, especially under the restrictions imposed by COVID-19.
3. Flyers with detailed information together with Bti fizzy tablets for self-help were distributed to 1820 households on 11 and 12 May 2020.
4. Deployment of 30 ovitraps (approx.1 trap/2 ha) across the district that were inspected at 2-week intervals (Fig. 2). Eggs were counted and checked for embryogenesis according to the description in section "Quality control of the sterile* Ae. albopictus* males”.
5. DtD activities from May to September 2020 in five rounds to ensure the action was effective and cost-efficient. The first round was from 18 May to 7 June during which time all properties were visited and inspected, and breeding sites were carefully mapped and treated with Bti. The data were entered into Q-GIS platform and used to support subsequent control measures by placing the focus on breeding site hotspots and the number of eggs in the ovitraps. The second round (23 June to 7 July) consisted of treating the hotspots on all properties where breeding sites of *Ae. albopictus* were recorded during the first round. The third round (28–31 July) consisted of treating the breeding site hotspots on properties within a 100-m radius of the six ovitraps (sites 2A, 4A, 5A, 1B, 4B, 7B on Fig. 2) in which eggs of *Ae. albopictus* were found. The fourth round (17–21 August) was similar to the second round, with inspections and treatments of properties with rainwater containers and small breeding sites. The fifth round (14–22 September) was similar to the first round: all houses were re-inspected and breeding sites were checked for larvae and treated if needed. Residents who agreed to participate were asked to complete a questionnaire on their own contribution to combat Asian tiger mosquitoes and their view of the success of the campaign.

In Metzgergrün, from June to October 2020, accessible gardens were inspected at 4-week intervals and treated with Vectobac WG in five rounds.

### Integration of SIT

The third pillar of the CP–DtD–SIT management strategy was to challenge *Ae. albopictus* populations by releasing sterile males under field conditions. This included the logistics of providing a steady supply of sterile males, quality control and the effect of releasing sterile male mosquitoes on field populations.

In this context, *Ae. albopictus* eggs collected in Heidelberg in 2017 were used to start a mass-rearing program at the Centro Agricoltura Ambiente “G. Nicoli” (CAA) in Crevalcore, Italy. This approach was chosen to prevent the use of mosquitoes with different genetic backgrounds. Following approval by regulatory authorities, the laboratory-reared mosquitoes were shipped from Italy to Germany.

#### Mass production of *Ae. albopictus*

An effective mass-rearing technique is essential to ensure the sustainable large-scale production of high-quality sterile males [26–29]. In our program, the mass-rearing methods developed by the Insect Pest Control Laboratory of the FAO/IAEA were adopted [27–31].

Sex sorting was performed by using Fay-Morlan glass sorters at the pupal stage in water [32]. This technique exploits the difference in size between male and female pupae. The aim was to keep the number of residual females to no more than 1% of the released sterile males.

#### Sterilization by irradiation of pupae

Pupal irradiation was conducted at the Medical Physics Department of St. Anna Hospital (Ferrara, Italy), using an IBL 437 irradiator (CIS Bio International, Bagnols-sur-Cèze, France) with a Cs-137 linear gamma-ray source. Male pupae aged 24–32 h in water [22] were exposed to a dose of 35 Gy (1.85 Gy/min for 19 min). This dosage provides the optimal combination between male sterility and competitiveness [22, 33].

After irradiation, the pupae were kept in a room maintained at 28 ± 1 °C for adult emergence. The young adults were chilled at 8–10 °C and packaged for delivery to the field site by DHL flight express service. The time span between leaving the production facility and field release was always in the range of 20 to 24 h.

#### Quality control of the sterile* Ae. albopictus* males

Thirty sterile males, randomly sampled from three different batches (SIT batches: 7, 8, 9; see Table 1), were individually released into each of three BugDorm rearing cages (BioQuip, Compton, CA, USA). Then, 30 virgin *Ae. albopictus* females from our colony (Heidelberg strain) were introduced into each cage and the cages kept at 252 °C and 70 ± 5% RH under an 8/16-h (dark/light) light regimen. Three cylindrical dark containers (diameter: 7 cm; height: 6 cm) were positioned in each cage, each half filled with water and containing a wooden board (length: 8 cm; width: 3 cm) as a support for oviposition and egg collection. In addition, a receptacle with cotton, soaked with a 10% sugar solution, 10 raisins and a piece of apple were provided as a carbohydrate source. Three cages with the same number of unirradiated males and females were included as control. At 24, 48, 72, 96, 120 and 144 h, the forearm of the principal investigator (PI) was exposed in each cage for 20 min to allow the females to take an ad libitum blood meal. The number of biting females per offering session was recorded. The females were kept for another 6 days in the cages for oviposition, following which time the wooden boards were removed and kept for 5 days in chambers with wet cotton (> 90% RH) to allow complete embryogenesis. The wooden boards were then transferred into a hatching container (size: 22 × 7 × 4.5 cm) and flooded with tap water. The hatched larvae were counted after 24 h and removed from the container. Then, containers with wooden boards were filled with a 10% hydrogen peroxide solution and kept for 48 h at 25 ± 2 °C to bleach the exochoria [34]. The boards were removed, and all eggs (including the egg-shells of the hatched larvae) were counted using a binocular microscope (model SMZ-171; Motic Deutschland GmbH, Wetzlar, Germany) and the embryogenesis of each single egg was assessed. Due to the resulting transparency of the exochorion in eggs with fully developed embryos, the eyes of the embryo and the “hatching tooth” could be easily recognized as dark spots on the head capsule at the anterior part of the embryo. It cannot be excluded that some of the developed embryos suffered from chromosomal damage, which does not allow hatching or normal development; thus, sterility may had been underestimated. Eggs showing no embryonic structures were rated as “sterile.” Sterility was also tested by disrupting or bursting the egg-shell with a needle to identify the segmentation of an existing embryo or non-segmented whitish egg masses. Non-embryonated egg-shells burst easily when touched with the needle.

**Table 1:** Overview of the sterile male Asian tiger mosquitoes released in Ludwigshafen (Melm) and Freiburg (Metzgergrün) in 2020

Batch no.	Speyer: no. of males	No. of males per container	Corrected no. of males	CAA: percentage of irradiated females (%)	Control (IfD): percentage of irradiated females	Freiburg: no. of males	Time of release (in 2020)	Mortality rate after shipment (%)
1	12,000	–	11,628	1.65	–	–	29 May	n.a
2	22,000	–	21,318	1.6	–	–	09 June	n.a
3	19,000	875	18,411	2.45	2.31	–	17 June	n.a
4	17,000	1070	16,473	1.75	2.42	–	30 June	n.a
5	13,000	865	12,597	1.54	1.15	–	07 July	1,4
6	19,000	968	18,411	0.22	1.5	5000	15 July	8
7	12,000	943	11,628	0.35	0.1	5000	21 July	4.3
8	24,000	893	23,256	1.53	1.34	5000	28 July	30
9	20,000	930	19,380	0.7	0.86	8000	04 August	10
10	29,000	1050	28,101	0.62	0.99	8000	11 August	n.a
11	21,000	968	20,349	1.34	1.34	8000	18 August	0.5
12	16,000	933	15,504	0.78	1.18	12,000	25 August	0.5
13	9,000	969	8,721	1.64	1.32	7000	01 September	5
14	17,000	1173	16,473	0.74	0.34	12,000	08 September	n.a
15	18,000	1065	17,442	0.55	0.47	17,000	15 September	n.a
16	14,000	980	13,566	0.37	0.81	18,000	22 September	n.a
17	17,000	875	16,473	0.72	1.71	12,000	29 September	n.a
18	21,000	944	20,349	1.82	1.34	19,000	07 October	n.a
Total	320,000	mean± SD: 969 ± 84	310,080	mean ± SD: 1.13 ± 0.64%	mean ± SD: 1.19 ± 0.63%	136,000		8.5 ± 10%

CAA, Centro Agricoltura Ambiente “G. Nicoli”, Crevalcore, Italy; IfD, Institute of Dipterology, Speyer, Germany; n.a., information not available; SD, standard deviation

Data in table also include the number of irradiated females determined by the IfD (Control), the number of females per batch according to CAA and IfD and the mortality rate of irradiated males after shipment

#### Effect of the ratio between sterile and fertile males in cage experiments

In general, the implementation of standard control measures aimed at reducing wild *Ae. albopictus* populations is essential to increase the efficacy of SIT in mosquito management programs. The dose of radiation is chosen so as to damage the sperm while only minimally affecting the somatic cells of the sterile males [22] in order not to significantly reduce the competitiveness of the sterile males in the mating process. Thus, we tested the effect of different ratios of wild males versus sterile males using the same experimental design as in the test series for quality control. In BugDorm cages (size: 30 × 30 × 30 cm) (BioQuip), 30 females per cage were challenged with the following ratios of wild to sterile males: (i) 1:1 (15 wild:15 sterile males); (ii) 1:5 (5 wild: 25 sterile males); and (iii) 1:10 (3 wild:30 sterile males). In each cage, we placed three dark cylindrical containers (diameter: 7 cm; height: 6 cm) half-filled with water and containing a wooden board for oviposition. The same carbohydrate source as used in the previous series was offered; similarly, after 24, 48, 72, 96 120 and 144 h, an ad libitum blood meal was offered by exposing the forearm of the PI. The number of biting females per blood feeding session was recorded. After the last blood meal, the females were kept for another 6 days in the rearing cages to allow oviposition, following which the wooden boards were removed, marked and kept for another 5 days in chambers with wet cotton (> 90% RH) to allow complete embryogenesis. The rate of embryogenesis was assessed as described the preceding section. Each trial was conducted in three replicates.

#### Shipment of sterile* Ae. albopictus *males

The shipment of sterile males in mosquito management programs has to be cost-effective and timely, with mortality rates of the caged males being as low as possible. In the course of our study, we tested small round plastic containers (diameter: 5.2 cm; height: 4.7 cm) fitted with a dark plastic lid as shipment containers. Each container holds about 1000 radiated males. These plastic containers were packed in a second plastic container that in turn was packed inside a styrofoam box (size: 49 × 36 × 36 cm, volume: 63.5 l) that contained 11 gel packs as cooling elements (Blue Ice; DrycePharma, Cernusco sul Naviglio, Italy; www.dryce-pharma.com) and two frozen gel packs (Green Ice; DrycePharma) to keep the temperature in the styrofoam box at approximately 10 ± 2 °C. The frozen cooling elements were in bubble wrap to avoid direct contact with the containers holding the sterile males. According to the size of the area to be treated, the styrofoam box contained up to 30 small plastic containers. The styrofoam boxes were shipped on a weekly basis from May until October by DHL on an overnight service. The cost of each shipment with DHL was recorded.

#### Assessment of the accurate number of the sterile males and females per container and shipment

Of the 18 shipments sent from CAA, Italy, one plastic container holding approximately 1000 radiated males was transferred to a refrigerator and kept for 2 h at − 15 °C to kill all mosquitoes. The number of males and females per container was determined under a binnocular microscope (model SMZ-171; MoticDeutschland GmbH), which allowed determination of the approximate number of released males. Furthermore, it was our goal to keep the contamination of the samples with *Aedes* females as low as possible with the appropriate use of the sexing technique [32]. We find that 1% females per small container is acceptable in terms of their not contributing to a nuisance situation caused by released *Aedes* females even if fully sterile.

#### Field application of SIT

The sterile males were released within trial area B (Melm, city of Ludwigshafen) after implementation of standard interventions by CP and DtD control. All releases took place during the early evening (7 p.m.) at 13 sites (Fig. 2; Table 1). Eighteen recurrent weekly releases between 29 May and 7 October 2020 were carried out, with a total of > 310,000 sterile males released. This corresponds to a mean number of 1013 sterile males released per week per hectare within the 17-ha trial area.

In Metzgergrün, 136,000 sterile males were released across the 4.5-ha site from 15 June to 7 October 2020, which amounts to a total of 2320 sterile males per hectare (Table 1; Fig. 3). This relatively higher number of released sterile males was chosen because of the larger wild population of *Ae. albopictus* in the Metzgergrün district compared to the Melm district in Ludwigshafen.

### Assessment of the efficacy of the implemented control strategy

Surveillance of the *Ae. albopictus* population was based on inspection of the breeding sites, including larval sampling, egg counts and embryogenesis checks in ovitraps.

Larval breeding sites that contained water were inspected for mosquitoes by aid of a torch, and samples were collected with a plankton net and identified to the species level [35].

Standard ovitraps to determine the number of deposited eggs were the main tool to assess the effect of the intervention, also also allowed an estimate of the population density [36]. The ovitraps consist of a dark plastic container with a total volume of 1.5 l. They were positioned on the ground or hung in shaded sites at a maximum height of 1.5 m and filled three quarters full with hay infusion (3 g hay pellets dissolved in 5 l of tap water). A wooden board (length: 17 cm; width: 3 cm) was placed in the ovitrap to support oviposition. To prevent the development of larvae to adults, 10 granules of Vectobac® G (activity: 200 ITU/mg) were added to the water. The wooden boards were replaced at 2-week intervals and the water replenished. The boards were marked with the date and site of collection, wrapped in paper foil and stored at room temperature or, on rare occasions, in a refrigerator until the egg count. Random morphological determination was done by hatching some of the eggs and rearing them to the fourth-larval instar [37, 35]. Sterile eggs were identified as described above.

### Deployment of the ovitraps in the test areas

1. Melm (Ludwigshafen): Starting on 18 May 30 ovitraps were evenly positioned across the 65-ha area, 10 in each of the three sectors (A, B, C) (Fig. 2). The wooden boards with the eggs were collected at 2-week intervals between 1 June and 5 October 2020. The number of eggs and embryogenesis were assessed in previous sections.
2. Metzgergrün (Freiburg): Across the 4.5-ha test area, 21 ovitraps were positioned as described above. This district was heavily infested with *Ae. albopictus* and therefore chosen as the SIT test area. Following DtD control and application of Bti (Vectobac® WG), the release of sterile males started in middle of July (Fig. 3).
3. Gartenstadt (Freiburg): This district was chosen as the control area without SIT application. It was similarly infested by *Ae. albopictus* as Metzgergrün. Eighteen ovitraps were installed. The wooden boards were collected at 2-week intervals between 15 August and 13 October. The number of eggs and the sterility were assessed as described above.

### Statistical analyses

For statistical analyses, Student’s t-test (two samples assuming unequal variances) was applied to the quality control of sterile males and to the cage experiments to assess the effect of the ratio of sterile to fertile males as well as to assess the effect on the sterility in the field (Microsoft Excel, version 16.45.21011103; Microsoft Corp., Redmond, WA, USA).

## Results

### Community participation

A total of 1820 households received written information on the control of *Ae. albopictus* and fizzy Bti tablets in their mailboxes (time for the distribution per household: < 1 min). The positive effect of the active cooperation of the residents is documented by the high rate of permission to access houses during the first DtD round at the end of May/early June, with access granted by 78.4% of households; only 9.1% of household refused entry to their properties, while 12.5% were absent. More than half of the properties (55.4%) did not contain any breeding sites (Table 2). During the questionnaire survey in September, of the 517 property owners who responded, 298 stated that they had implemented the control actions proposed on the flyer (57.6%), while 28 people (5.4%) claimed that the control of the Tiger mosquito is not important for them.

**Table 2:** Overview of the community participation pillar of the mosquito control program

CP pillar	Round 1	Round 2	Round 3	Round 4	Round 5
Date	18 May to 12 June 2020	23 June to 7 July 2020	27–31 July 2020	17–21 August 2020	14–22 September 2020
Total no. of properties	953	318	110	273	1029
Entered,* n* (%)	747 (78.38%)	261 (82.07%)	110	193 (70.7%)	724 (70.36%)
Absent,* n* (%)	119 (12.49%)	23 (7.23%)	–	71 (26%)	205 (19.92%)
Refused,* n* (%)	87 (9.13%)	34 (10.69%)	–	9 (3.3%)	100 (9.72%)
No. of properties with breeding sites	333 (44.58%)	240 (91.95%)	–	147 (76.17%)	517 (71.4%)
No. of properties without breeding sites	414 (55.42%)	21 (8.05%)	–	46 (23.83%)	207 (28.6%)
No. of potential breeding sites (all)	1535	741	340	1044	2668
No. of positive breeding sites (all)	6 (0.39%)	No larvae	15 (4.41%)	26 (2.49%)	82 (3.07%)
No. of positive breeding sites for *Ae. albopictus*	1 (0.07%)	No larvae	3 (0.8%)	1 (0.1%)	13 (0.49%)
CI total (%)	0.39%	–	4.41%	2.49%	3.07%
CI *Aedes albopictus*, %	0.07%	–	0.88%	0.1%	0.49%
Working hours,* n*	540	140	84	80	325
Bti comsumption	4	2	1	3	7.3

Data on accessibility of the properties, occurrence of breeding sites and infestation rates with mosquito developmental stages, Bti consumption (Vectobac WG) in kg and number of working hours during the five rounds of control in Melm district of Ludwigshafen are provided

Bti * Bacillus thuringiensis israelensis*; CI, Container Index

### DtD control and Bti treatments

#### Assessment of the optimum dosage of Bti when applied with pressurized hand sprayers

**Fig. 4:**
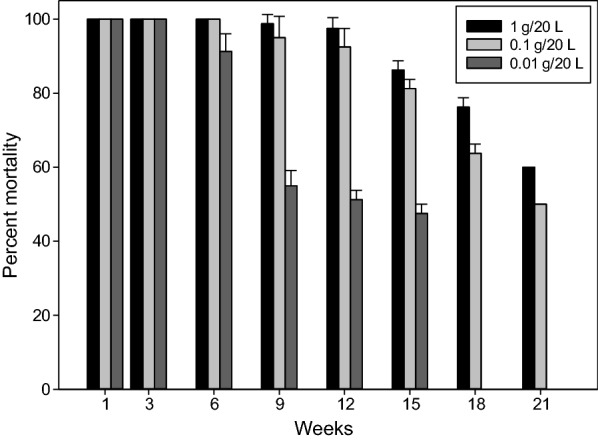
Long-term effect of Vectobac WG on *Aedes albopictus* larvae when high dosages were applied in the larger water containers

Water containers were treated with a dose of 1 g Vectobac® WG per 20 l (3 × 10^6^ ITU). The excellent persistence of mosquitocidal activity is shown in Fig. 4. Mortality of 100% was achieved during 8 weeks, followed by a small decrease (of 1%) over the next 4 weeks. Only after 15 weeks did mortality start to drop to < 90%. These results were obtained with a weekly exchange of 25% of the water to simulate the use of collected rainwater to irrigate gardens.

**Fig. 5:**
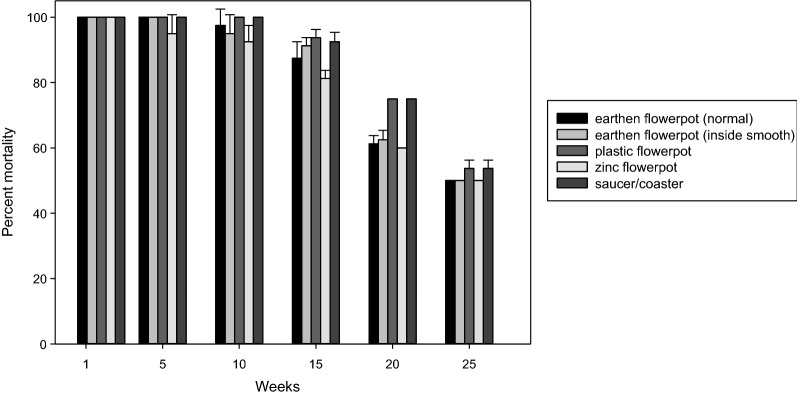
Effect of Vectobac WG at a dosage of 2.5 g per small container on third-instar larvae of *Ae. albopictus* in different types of flower pots and flower pot saucers

Larval control of *Ae. albopictus* in small containers was achieved for > 1 month with an initial application rate of 7.5 × 10^6^ ITU per container) (Fig. 5).

#### Efficacy of the DtD treatments in Melm district (city of Ludwigshafen)

During the five DtD rounds, including two complete rounds (rounds 1 and 5) and three rounds focusing on mosquito hotspots (rounds 2–4) on properties and their surroundings when positive ovitraps or mass breeding sites were observed, a total of 2683 properties were inspected. Detailed results are given in Table 2. Of the 2683 properties, permission was granted to enter 2035 (75.9%), 418 (15.6%) owners were absent and 230 (8.6%) owners refused permission to enter. A total of 6328 breeding sites, including 83 rainwater containers, were treated with a high Bti dosage to achieve a long-lasting effect; the data were recorded in the Q-GIS platform. Of these 6328 breeding sites, 129 were infested with mosquito larvae (CI: 2%) and 18 were infested with *Ae. albopictus* (CI: 0.3%). The highest CI values were recorded at the end of August, with a CI of 4.4% for all mosquitoes and 0.9% for *Ae. albopictus* (Table 2). The positive effect of the control strategy is demonstrated by the CI of 0.5% for *Ae. albopictus* at the end of September. A total of 1169 working hours (across 2–6 inspectors) was invested in the second pillar (DtD strategy) of the control program, which results in an inspection time per property of 26 min. Altogether, 17.3 kg of Vectobac WG was applied to the 2683 mosquito breeding sites.

#### Efficacy of routine DtD treatments in Metzgergrün (city of Freiburg)

The routine treatments in Metzgergrün (Freiburg) were performed over five rounds of DtD activities and Bti treatments. Starting in the second half of June, the routine treatments were conducted at approximately 4-week intervals. Bti application was carried out until all properties were treated, a process which took 5 consecutive days. After each application round, the accessibility of the area was evaluated and recorded. Compared to other areas in the city of Freiburg, the accessibility of the properties was classified as ranging from poor to moderate. Throughout the season, residents were provided with blister packages of fizzy Bti tablets for self-help, including those whose properties could not be inspected.

### Application of SIT against *Ae. albopictus*

#### Laboratory evaluation of the SIT

##### Quality control of sterile *Ae. albopictus* males

The mean (± standard deviation) egg sterility of females inseminated by sterile *Ae. albopictus* males was 87.5 ± 9.2%, whereas the sterility of eggs derived from non-irradiated males and females was only 3.3 ± 2.8% (Table 3). The true sterility might be even higher because the bleaching method may fail to detect embryos entering the stage of delayed mortality (data not published). According to Student’s t-test, all results are significant (Student’s t-test: *P* ≤ 0.001). To the contrary, 96.7 ± 2.8% of the eggs derived from non-irradiated individuals were embryonated, while only 12.5 ± 6.5% of the eggs laid by females inseminated by irradiated males developed into embryos (Table 3).

**Table 3:** Assessment of the sterility and number of eggs laid by radiated males versus wild-type males

Cage	Wild-type cages			SIT cages		
	No. of eggs	Embryonation^a^	Sterility^b^	No. of eggs	Embryonation^a^	Sterility^b^
Cage 1	490	459 (93.67%)	31 (6.33%)	944	54 (5.73%)	890 (94.27%)
Cage 2	1000	992 (99.2%)	8 (0.80%)	939	122 (13%)	817 (87%)
Cage 3	544	529 (97.24%)	15 (2.76%)	707	132 (18.67%)	575 (81.33%)
Mean ± SD		96.70 ± 2.8%	3.3 ± 2.8%		12.47 ± 6.49%	87.53 ± 9.15%

SIT, Sterile insect technique

^a^Reported as the number (%) of embyronated eggs from non-irradiated (wild-type cages) and irradiated (SIT cages) individuals, respectively

^b^Reported as the number (%) of sterile eggs derived from non-irradiated males and females (wild-type cages) and from females inseminated by sterile *Ae. albopictus* males (SIT cages), respectively

##### Accuracy in sex sorting

The number of irradiated *Aedes* females in the delivered batches slightly exceeded 1%. The percentage of irradiated females recorded by CAA was 1.1 ± 0.6% and is almost identical to the percentage (1.2 ± 0.6%) evaluated by IfD (Table 1). These data indicate a release of about 3700 irradiated females in Melm district (Ludwigshafen) and 1620 radiated females in Metzgergrün (Freiburg).

##### Determination of the most effective ratio between sterile and fertile males in cage experiments

**Fig. 6:**
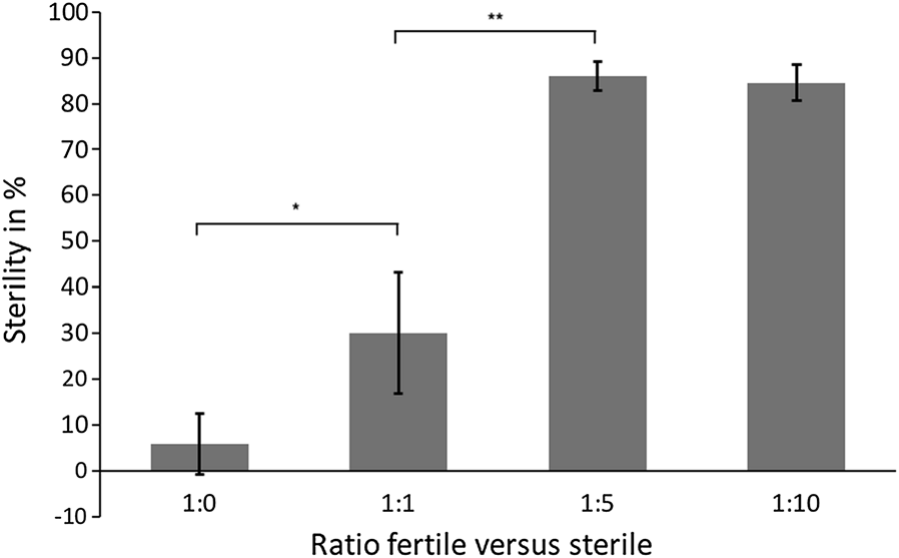
Effect of the ratio between radiated and unirradiated males on the egg sterility rate. Asterisks indicated statistically significant differences according to Student’s t-test at *P ≤ 0.05, ** *P* ≤ 0.01

The results of this experiment document the importance of determining the ratio between wild-type and irradiated males (Fig. 6; Additional file 1: Table S1). The egg sterility of the non-irradiated populations is 5.9 ± 6.6%. When the ratio of radiated to non-irradiated males is 1:1 (15 radiated:15 non-irradiated males per cage), the sterility is 30.1 ± 13.1%, but when the ratio is 1:5 (5 fertile males:25 sterile males, i.e. overflooding ratio), the sterility increases to 85.9 ± 3.2%; these results are highly significant according to the t-test ( *t*_6_ = 2.92, *P* = 0.0095). At a ratio of 1:10 (3 fertile males:30 sterile males) the sterility remained at a nearly unchanged level of 84.6 ± 4.0%.

#### Field application of SIT

##### Release of sterile *Ae. albopictus* males

A total of 310,000 irradiated males were sent to Ludwigshafen (Melm) in 18 shipments, while a total of 136,000 males were sent in 13 shipments to Freiburg (Metzgergrün) by DHL overnight services (Table 1). All shipments reached their respective destination within 24 h. The mortality rate of the irradiated males during transport averaged 8.5 ± 10.1%.

Table 1 provides the exact numbers of released sterile males, which amounted to the release of about 1000 sterile males per hectare on a weekly basis from 29 May to 7 October in Melm district (Ludwigshafen) and of 2300 sterile males per hectare from 15 July to 7 October in Metzgergrün (Freiburg).

##### Assessment of the efficacy of the implemented integrated control strategy

- Melm district (city of Ludwigshafen)
  Of the 30 installed ovitraps, 919 eggs of *Ae. albopictus* were found in 17 ovitraps from 15 June to 7 September 2020 (Table 4; Additional file 2: Table S2). The mean number *of Ae. albopictus* eggs per trap per 2 weeks was 4.4 eggs. The highest number of eggs per trap was 110 eggs on 13 July in trap 2A (Fig. 2). The highest number of positive traps per sample date occurred on 24 August, with 10 traps being positive with a total of 301 eggs (Additional file 2: Table S2). The traps collected on 29 May, 21 September and 5 October did not contain eggs.

**Table 4:** Number of *Aedes albopictus* eggs and percentage of sterility in eggs in ovitraps located in Melm (Ludwigshafen)

Variables	Collection date (in 2020)						Total
	15 June	29 June	13 July	10 August	24 August	7 September	
Total no. of eggs	45	88	216	173	301	96	919
Section A							
Total no. of eggs	45	64	120	8	34	3	274
No. of sterile eggs	2	6	103	0	6	3	120
Sterility, % (mean ± SD)	4.44%	9.37%	85.83%	0	17.65%	100%	36.21 ± 40.65%
Section B (SIT)							
Total no. of eggs	0	24	91	101	114	90	420
No. of sterile eggs	0	23	59	84	91	90	347
Sterility, %	0	95.83%	64.84%	83.17%	79.82%	100%	84.73 ± 12.48%
Section C							
Total no. of eggs	0	0	0	64	153	3	220
No. of sterile eggs	0	0	0	8	74	3	85
Sterility, % (mean ± SD)	0	0	0	12.5%	48.37%	100%	53.62 ± 35.91%

**Fig. 7:**
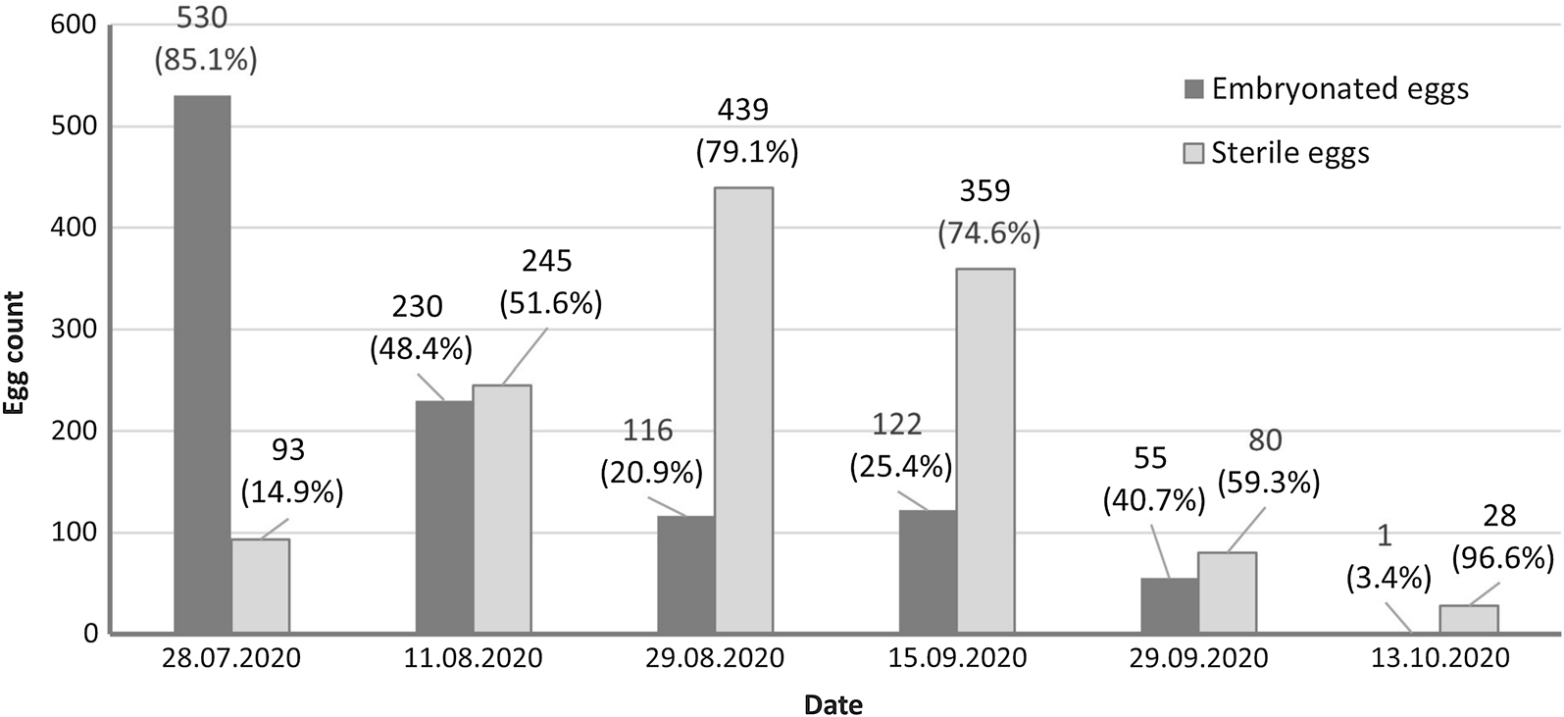
Sterility of *Ae. albopictus* eggs in the SIT area of Metzgergrün
  In the control area (without SIT) (Gartenstadt, Freiburg) on the collection dates from 29th of August to the 13th of October (4 × bi-weekly sampling), 1615 eggs were collected (Table 5; Additional file 4: Table S4). The mean sterility amounted to 14.6 ± 7.3%. In the first half of September, random breeding sites were inspected for *Ae. albopictus* larvae in a designated part of the control area. The samples were taken from around 35 different properties covering an area of 4 ha; of the 105 breeding sites inspected, 29 contained *Ae. albopictus* in various developmental stages, resulting in a CI_albo_ of 27.7%.

The weekly release of about 1000 sterile males per hectare from early May until early October in area B (CP + DtD + SIT) resulted in an overall sterility of 84.7 ± 12.5% (Table 4; Additional file 2: Table S2), which was significantly higher than that in area A (CP + DtD only) (t-test:* t*_9_ = − 1.83, *P* = 0.05). Of the 274 eggs collected in section A, 36.2 ± 40.7% (mean ± SD) were sterile; of the 220 eggs collected in section C (CP + DtD), 53.6 ± 35.9% (mean ± SD) were sterile. The relatively high sterility in the adjacent non-SIT-treated sections A and C can be explained by the migration of sterile males and/or females which mated with sterile males into the non-SIT control areas. The absence of eggs in any of the ovitraps after 7 September indicates the successful control strategy.
  In the second half of September, 2668 breeding sites were inspected, and only 13 of these contained *Ae. albopictus* larvae, resulting in a very low CI_albo_ of 0.5%.
- Metzgergrün and Gartenstadt (city of Freiburg)
  A total of 2298 eggs were collected in bi-weekly collections from the 21 ovitraps employed in the SIT area of Metzgergrün (Freiburg) during the sampling period (14 July to 13 October; first collection date: 28 July) (Table 5). This amounts to 18.23 eggs per trap per 2-week collection intervals and is about fivefold higher than the number of eggs collected in almost the same time period in the SIT area in Ludwigshafen. The release of about 2320 sterile males per hectare on a weekly basis from 15 July to 7 October produced sterility rates that increased from 14.9% on 28 July to 96.6% on 13 October (Fig. 7; Additional file 3: Table S3). The mean sterility rate was 62.7 ± 25.8% (Table 5).

**Table 5:** Number of *Ae. albopictus* eggs and percentage of sterility of the eggs in the SIT area (Metzgergrün) and the control area (Gartenstadt)

Variables	Collection date (in 2020)						Total
	28 July	11 August	29 August	15 September	29 September	13 October	
SIT area (Metzgergrün)							
No. of eggs	623	475	555	481	135	29	2,298
No. of sterile eggs	93	345	439	359	80	28	1,344
Sterility, % (mean ± SD)	14.9%	51.6%	79.1%	74.6%	59.3%	96.6%	62.68 ± 25.75%
Control area (Gartenstadt)							
No. of eggs	–	–	498	902	80	135	1,615
No. of sterile eggs	–	–	132	129	8	10	279
Sterility, % (mean ± SD)	–	–	26.5%	14.3%	10.0%	7.41%	14.55 ± 7.32%

### Cost analyses

The cost analyses reported here are based on a precise evaluation of the working time and utilization of materials in the Melm district (Ludwigshafen), in relation to the number of inhabitants as well as the number of inspected and treated properties (Table 2). The district of Melm comprises 65 ha, with 1029 properties and approximately 2800 residents.

#### CP based on distribution of flyers and fizzy Bti tablets

Costs for 1100 flyers: 33 euros.

Costs for 1820 Bti-blisters each with 10 fizzy Bti tablets for self-help: 5096 euros.

Costs for distribution: 25 h, each at 17.00 euros per hour, for a total of 425 euros.

**Total costs**: 5521 euros.

(Costs per property: 5.37 euros for one application at the beginning of the season; costs per person: 1.97 euros.)

#### DtD activities (Table 2)

Rounds 1–5:

2683 properties; number of breeding sites controlled: 6328.

Working hours: 1169 h, each at 17 euros, for a total of 19,873 euros (26 min per property).

Bti consumption: 17.3 kg of Vectobac® WG: 657.40 euros.

Consumables: 100 euros.

**Total costs of rounds 1–5 of DtD**: 20,630 euros.

(Costs per property: 7.70 euros; costs per person: 7.37 euros.)

Round 5 (field worker were more experienced in round 5 compared to the previous rounds):

1029 properties; no. of breeding sites controlled: 2668.

Working hours: 325 h, each at 17 euros, for a total of 5525 euros (19 min per property).

Bti consumption: (7.3 kg of Vectobac® WG): 277 euros.

Consumables: 100 euros.

**Total costs of round 5 of DtD**: 5902 euros.

(Costs per property: 5.74 euros; costs per person: 2.11 euros.)

#### SIT (in sector B [Melm district, city of Ludwigshafen])

272 properties; 17 ha.

Total no. of releases: 18; no. of released sterile males: 310,000; approximately1000 males per hectare per week.

Costs for sterile males: 18,880 euros.

Costs for DHL shipments (18 × 190 euros): 3420 euros.

Working hours per release 2 h = 36 h: 612 Euros.

**Total costs for SIT in sector B (Melm district)**: 22,912 euros.

(Costs per ha: 1348 euros; costs per property: 84 euros).

The higher costs are explained by the experimental status of the project.

#### Monitoring the 30 ovitraps employed in the test area in Ludwigshafen

Costs for positioning: 15 working hours: 255 euros.

Costs for collecting the wooden boards (10 × 2 h): 340 euros.

Costs for evaluation (30 boards × 10  × 15 min): 75 h × 17 euros: 1275 euros.

Consumables: 75 euros.

**Total costs for monitoring**: 1945 euros.

## Discussion

The goal of this large-scale integrated field trial was the maximum reduction or even the elimination of established populations of *Ae. albopictus*. The first two pillars of the program comprised CP and DtD control by trained staff with long lasting Bti-treatment, and these were supplemented with SIT as the third pillar. CP and DtD are important to reduce *Ae. albopictus* in obvious and easily accessible breeding places. Sterile males are able to find remaining females, such as those emerging from cryptic breeding sites. The selected trial sites, all urban environments infested by *Ae. albopictus* in the cities of Ludwigshafen and Freiburg, were ideal for the three-pillar approach. The majority of *Ae. albopictus* breeding sites are located on private properties, and it is therefore in the interest of residents to eliminate breeding sites of *Ae. albopictus* in close proximity to their property. With proper instruction and using Bti, collaboration with residents within the framework of an integrated control program is cost-effective and sustainable [38–41]. The DtD activities conducted by trained staff proved to be the backbone of the integrated control approach. However, there is room for improvement in terms of cooperation between the residents and DtD teams: average accessibility was 80% whereas the target was a minimum access of 95% [21] (Table 2). The mean costs involved in the control measures by CP and DtD activities amounted to approximately 13 euros per property, which is about sevenfold less than the costs for applying SIT.

A program aimed at controlling the container-breeding *Ae. albopictus* requires a long-lasting larvicide to make it cost effective and efficient. This goal was achieved with increased doses of Bti [42]. The results obtained in Ludwigshafen were convincing. As a result of the stringent Bti application, the CI of *Ae. albopictus* was pushed down from 10.9% to < 1% (Table 2) and, in contrast to the previous year, residents did not report any mosquito nuisance. This decrease in the *Ae. albopictus* population is also confirmed by the low number of eggs found in the ovitraps in the Melm area (mean number eggs per ovitrap per week: 4.3). In comparison, in the control area of Gartenstadt (Freiburg), six ovitraps contained considerably more than 100 eggs per ovitrap and a maximum of 340 eggs per ovitrap per 2 weeks. Carrieri et al. [43] calculated the epidemic risk threshold for chikungunya transmission based on the mean egg density per trap per week, reporting that infections can already occur when the average number of eggs per trap per week exceeds 44 eggs; this threshold was exceeded in the control area of Gartenstadt. Climate change with rising temperature will very likely accentuate this problem.

Risk–benefit parameters do not apply to the integrated approach for the control of *Ae. albopictus*. The use of Bti even in higher doses is safe, and the release of sterile males is not associated with any negative impact [44–47]. Special consideration should be given to the cost–benefit analysis reported here. In general, the costs exist in an up-front investment to prevent both the spread and high density of *Ae. albopictus* populations. Protection from autochthonous viral infections must have the highest priority. Thus, the costs of our strategy, which is aimed at protecting residents from *Ae*. *albopictus*, amount to an estimated 9.5 euros per person per season. An in-depth cost–benefit analysis is of high importance, should this large-scale pilot study based on the three pillars, with SIT as the key element, become a standard approach to the control of *Ae. albopictus*. An integrated control program of *Ae. albopictus* is only acceptable in the long run if the benefits outweigh the costs. The calculation and compiling of the costs are relatively straightforward. Based on our experience fixed and variable costs will be reduced with experience, routine and volume, which applies to the production of sterile males. The benefits are harder to calculate. There are two main parameters to consider: (i) the nuisance caused by *Ae. albopictus* and most important (ii) the prevention of autochthonous transmissions, especially of dengue, chikungunya and Zika virusus. The nuisance component relates to the quality of life when *Ae. albopictus* as an urban mosquito is widely established and people have to take personal protective measures. These measures include sprays and spirals/coils in particular. The costs per person in an *Ae. albopictus*-infested area may amount € 30 per family per season. (Antibrumm and/ Autan cost 10 euros per flask; spirals/coils cost CHF 10 per package). The reduction in quality of life is hard to estimate. However, there are many instances where people have to give up staying outdoors. A cost–benefit study conducted in the Upper Rhine Valley in 2008 revealed that residents were willing to pay an average of 3.50 euros per person per year [48].

Germany, like other countries north of the Alps, are at the beginning of the invasion by *Ae. albopictus* and thus confronted with a big challenge. Containment of *Ae. albopictus* will only succeed by close collaboration between all the stakeholders, from the residents who are directly affected to the field workers, researchers, up to government agencies. [49]. The importance and urgency to combat the Asian tiger mosquito has been recognized at all levels of the Federal Republic of Germany. Programs for surveillance, risk assessment and the evaluation of control tools are actively supported by the Federal Agency for Environmental Protection (UBA) in cooperation with the Friedrich Löffler Institute (FLI) and the Bernhard Nocht Institute (BNI). The State of Baden-Württemberg, which is currently most affected by *Ae. albopictus*, has financed risk assessment programs concerning the establishment of *Ae. albopictus* on a community level [19].

The toolkit to combat container-breeding mosquitoes includes powerful techniques and strategies such as CP, microbial control tools (e.g. Bti), biological control (e.g. using copepods, insect growth regulators [IGRs], surface layers), SIT (e.g. based on radiation), the insect incompatibility technique through the endosymbiont *Wolbachia-*induced incompatibility (IIT) and/or the combination of both SIT and IIT [21, 35, 50]. Last but not least, chemical products, such as methoprene, diflubenzuron, pyriproxyfen (all IGRs) or pyrethroids (adulticides), can be applied in case of outbreaks only. Space spraying of adulticides should be only applied in the case of health emergencies [51]. Modern genetic approaches, as in site-specific gene editing with CRISPR/Cas9, can augment the currently existing toolbox. Cas9-mediated gene editing can be an efficient platform for gene-driven strategies to introduce suppression and pathogen-blocking genes into wild mosquito populations [35, 52].

## Conclusions

This field pilot trial shows that an integrated control program can be most effective when it comprises three pillars, namely (i) CP, (ii) DtD activities, including long-lasting larviciding of all accessible larval sites with Bti to strongly reduce wild *Aedes albopictus* populations, as a precondition to the subsequent and successful application of SIT and (iii) SIT to strongly reduce or even eliminate *Ae. albopictus* populations. The combination of Bti and SIT, two highly effective, selective and safe measures, may achieve *Ae. albopictus* suppression without any negative impact(s) on public health and the environment.

## Supplementary Information

**Additional file 1: Table S1**. Effect of the ratio between radiated and unirradiated males on the egg sterility rate.

**Additional file 2: Table S2.** Number of* Aedes albopictus* eggs and percentage of sterility of the eggs in Melm (Ludwigshafen).

**Additional file 3: Table S3**. Number of* Aedes albopictus* eggs and percentage of sterility of the eggs in the SIT area (Metzgergrün).

**Additional file 4: Table S4**. Number of* Aedes albopictus* eggs and percentage of sterility of the eggs in the control area (Gartenstadt).

## Data Availability

All data generated or analyzed during this study are included in this published article and its supplementary information files.

## References

[1] Medlock JM, Hansford KM, Schaffner F, Versteirt V, Hendrickx G, Zeller H (2012). A review of the invasive mosquitoes in Europe: ecology, public health risks, and control options. Vector-Borne Zoonotic Dis.

[2] Becker N, Geier M, Balczun C, Bradersen U, Huber K, Kiel E (2013). Repeated introduction of *Aedes albopictus* into Germany. Parasitol Res.

[3] Becker N, Pluskota B, Kaiser A, Schaffner F, Mehlhorn H (2012). Exotic mosquitoes conquer the world. Parasitology research monographs 3.

[4] European Centre for Disease Prevention and Control (ECDC). Guideline for the surveillance of invasive mosquitoes in Europe. Stockholm: ECDC; 2012. https://www.ecdc.europa.eu/sites/default/files/media/en/publications/Publications/TER-Mosquito-surveillance-guidelines.pdf. Accessed 28 Dec 2021.

[5] European Centre for Disease Prevention and Control and European Food Safety Authority. Mosquito maps [internet]. Stockholm: ECDC; *Aedes albopictus*-current known distribution. 2021. https://ecdc.europe.eu/en/publications-aedes-albopictus-current-known-distribution. Accessed 28 Dec 2021.

[6] European Centre for Disease Prevention and Control. Autochthonous transmission of dengue virus in EU/EEA, 2010–2019. 2020. https://www.ecdc.europa.eu/en/all-topics-z/dengue/surveillance-and-disease-data/autochthonous-transmission-dengue-virus-eueea. Accessed 28 Dec 2021.

[7] European Centre for Disease Prevention and Control (EDCD). Autochthonous transmission of chikungunya virus in mainland EU/EEA, 2007–present. https://www.ecdc.europa.eu/en/all-topics-z/chikungunya-virus-disease/surveillance-threats-and-outbreaks/autochthonous. Accessed 28 Dec 2021.

[8] European Centre for Disease Prevention and Control (EDC). Epidemiological update: third case of locally acquired Zika virus disease in Hyères, France. 2020. https://www.ecdc.europa.eu/en/news-events/epidemiological-update-third-case-locally-acquired-zika-virus-diseasehyeres-france

[9] Dalla Pozza G, Majori G (1992). First record *of Aedes albopictus* establishment in Italy. J Am Mosq Control Assoc.

[10] Romi R (1994). *Aedes albopictus* in Italia: problemi sanitari, strategie di controllo e aggiornamento della distribuzione al 30 settembre 1994. Notiziario ISS.

[11] Eritja R, Palmer JRB, Roiz D (2017). Direct evidence of adult *Aedes albopictus* dispersal by car. Sci Rep.

[12] Angelini R, Finarelli AC, Angelini P, Po C, Petropulacos K, Silvi G, et al. Chikungunya in north-eastern Italy: a summing up of the outbreak. Euro Surveill. 2007;12(11):E071122.2. http://www.eurosurveillance.org/ViewArticle.aspx?ArticleId=3313. 10.2807/esw.12.47.03313-en 18053561

[13] World Health Organization (WHO) Outbreak news: Chikungunya, Spain. 2015. https://www.who.int/csr/don/10-august-2015-chikungunya/en/.

[14] Venturi G, Di Luca M, Fortuna C, Remoli ME, Flavia R, Severini F, et al. Detection of a chikungunya outbreak in central Italy, August to September 2017. Euro Surveill. 2017;22(39):28. 10.2807/1560-7917.ES.2017.22.39.17-00646. 10.2807/1560-7917.ES.2017.22.39.17-00646 PMC5709953 29019306

[15] European Centre for Disease Prevention and Control (ECDC). Cluster of autochthonous chikungunya cases in France – 23 August 2017. ECDC, Stockholm. 2017. https://www.ecdc.europa.eu/sites/default/files/documents/RRA-Chikungunya-France-revised-Aug-2017.pdf. Accessed 28 Dec 2021.

[16] Pluskota B, Storch V, Braunbeck T, Beck M, Becker N (2008). First record of *Stegomyia albopictus* (Skuse) (Diptera: Culicidae) in Germany. Eur Mosq Bull.

[17] Lühken R, Heitmann A, Jansen S, Schmidt-Chanasit J, Börstler J, Werner D (2020). Microsatellite typing of *Aedes albopictus* (Diptera: Culicidae) populations from Germany suggests regular introductions. Infect Genet Evol.

[18] Becker N, Schön S, Klein A-M, Ferstl I, Kizgin A, Tannich E (2017). First mass development of Aedes albopictus (Diptera: Culicidae)—its surveillance and control in Germany. Parasitol Res.

[19] Pluskota B, Augsten X, Kizgin A, Kühnlenz T, Stelzner L, Tokatlian Rodriguez A, et al. Aufbau eines standardisierten Kontrollkonzepts auf der Basis von Untersuchungen zur Effektivität von Monitoring- und Bekämpfungsmaßnahmen an einer Freilandpopulation der Asiatischen Tigermücke (*Aedes albopictus*) in Heidelberg. 2019. http://fachdokumente.lubw.baden-wuerttemberg.de.

[20] Zheng X, Zhang D, Li Y, Yang C, Wu Y, Liang X (2019). Incompatible and sterile insect techniques combined eliminate mosquitoes. Nature.

[21] Bellini R, Medici A, Puggioli A, Balestrino F, Carrieri M (2013). Pilot field trials with *Aedes albopictus* irradiated sterile males in Italian urban areas. J Med Entomol.

[22] Balestrino F, Medici A, Candini G, Carrieri M, Maccagnani B, Calvitti M (2010). Gamma ray dosimetry and mating capacity studies in the laboratory on *Aedes albopictus* males. J Med Entomol.

[23] Donati L, Carrieri M, Bellini R (2020). A door-to-door strategy for *Aedes albopictus* control in Northern Italy: efficacy, cost-analysis and public perception. Vector Biol J.

[24] Becker N (2002). Sterilization of *Bacillus thuringiensis israelensis* products by gamma radiation. J Am Mosq Control Assoc.

[25] Becker N (1997). Microbial control of mosquitoes: management of the Upper Rhine mosquito population as a model programme. Parasitol Today.

[26] Dame DA, Curtis CF, Benedict MQ (2009). Historical applications of induced sterilisation in field populations of mosquitoes. Malar J.

[27] Balestrino F, Puggioli A, Gilles JRL, Bellini R (2014). Validation of a new larval rearing unit for *Aedes albopictus* (Diptera: Culicidae) mass rearing. PLoS ONE.

[28] Balestrino F, Puggioli A, Bellini R, Petric D, Gilles JRL (2014). Mass production cage for *Aedes albopictus* (Diptera: Culicidae). J Med Entomol.

[29] Insect Pest Control Laboratory FAO–IAEA. Guidelines for mass-rearing of *Aedes* mosquitoes. Version 1.0. 2018. http://www-naweb.iaea.org/nafa/ipc/public/Guidelines-for-mass-rearingofAedes-osquitoes_v1.0.pdf.

[30] Puggioli A, Balestrino F, Damiens D, Lees RS, Soliban SM, Madakacherry O (2013). Efficiency of three diets for larval development in mass rearing *Aedes albopictus* (Diptera: Culicidae). J Med Entomol.

[31] Puggioli A, Carrieri M, Dindo ML, Medici A, Lees RS, Gilles JRL (2016). Development of *Aedes albopictus* (Diptera: Culicidae) larvae under different laboratory conditions. J Med Entomol.

[32] Focks DA (1980). An improved separator for the developmental stages, sexes, and species of mosquitoes (Diptera: Culicidae). J Med Entomol.

[33] Balestrino F, Puggioli A, Carrieri M, Bouyer J (2017). Bellini R Quality control methods for *Aedes albopictus* sterile male production. PLoS Negl Trop Dis.

[34] Hokama Y, Judson C (1963). A new bleaching technique with possible general use in entomology. Ann Entomol Soc Am.

[35] Becker N, Petrić D, Zgomba M, Boase C, Madon M, Dahl C (2020). Mosquitoes.

[36] Bellini R, Carrieri M, Burgio G, Bacchi M (1996). Efficacy of different ovitraps and binomial sampling in *Aedes albopictus* surveillance activity. J Am Mosq Control Assoc.

[37] Tanaka K, Mizusawa K, Saugstad ES. A revision of the adult and larval mosquitoes of Japan (including the Ryukyu Archipelago and the Ogasawara islands) and Korea (Diptera: Culicidae). Contr Am Entomol Inst Ann Harbor. 1979. pp. 987. https://agris.fao.org/agris-search/search.do?recordID=US8003808.

[38] Becker N, Djakaria S, Kaiser A, Zulhasril O, Ludwig HW (1991). Efficacy of a new tablet formulation of an asporogenous strain of *Bacillus thuringiensis israelensis* against larvae of *Aedes aegypti*. Bull Soc Vector Ecol.

[39] Becker N (1992). Community participation in the operational use of microbial control agents in mosquito control programs. Bull Soc Vector Ecol.

[40] Kroeger A, Dehlinger U, Burkhardt G, Anaya H, Becker N (1995). Community based dengue control in Columbia: people’s knowledge and practice and the potential contribution of the biological larvicide *B. thuringiensis israelensis* (*Bacillus thuringiensis israelensis*). Trop Med Parasitol.

[41] World Health Organization. Dengue guidelines for diagnosis, treatment, prevention and control: new edition. Special Programme for Research, Training in Tropical Diseases, Department of Control of Neglected Tropical Diseases, World Health Organization: Epidemic, & Pandemic Alert; 2009. pp.146. https://apps.who.int/iris/handle/10665/44188.

[42] Lacey LA (editor). Microbial control of insect and mite pests—from theory to practice. New York/Amsterdam: Academic press/Elsevier Inc. 2017.

[43] Carrieri M, Angelini P, Venturelli C, Maccagnani B, Bellini R (2012). Aedes albopictus (Diptera: Culicidae) population size survey in the 2007 chikungunya outbreak in Italy. II: estimating epidemic thresholds. J Med Entomol.

[44] World Health Organization (WHO). Microbial pest control agent *Bacillus thuringiensis*. In: Report of UNEP/ILO/WHO, Environ Hlth Criteria. WHO, Geneva. 1999: pp. 217.

[45] Lam PHY, Boon CS, Yng NY, Benjamin S (2010). *Aedes albopictus* control with spray application of *Bacillus thuringiensis israelensis*, strain AM 65–52. Southeast Asian J Trop Med Public Health.

[46] Pruszynski CA, Hribar LJ, Mickle R, Leal AL (2017). A large scale biorational approach using *Bacillus thuringiensis israeliensis* (strain AM65-52) for managing *Aedes aegypti* populations to prevent dengue, chikungunya and Zika transmission. PLoS ONE.

[47] Oliva CF, Jacquet M, Gilles J, Lemperière G, Maquart PO, Quilici S, et al.. The sterile insect technique for controlling populations of *Aedes albopictus* (Diptera: Culicidae) on Reunion Island: Mating vigour of sterilised males. PLoS One. 2012;7(11):e49414. 10.1371/journal.pone.0049414 PMC3504010 23185329

[48] Hirsch H, Becker N (2009). Cost-benefit analysis of mosquito control operations based on microbial control agents in the upper Rhine Vally (Germany). Eur Mosq Bull.

[49] Bellini R, Michaelakis A, Petrić D, Schaffner F, Alten B, Angelini P (2020). Practical management plan for invasive mosquito species in Europe: I. Asian tiger mosquito (Aedes albopictus). Travel Med Infect Dis.

[50] Kittayapong P, Ninphanomchai S, Limohpasmanee W, Chansang C, Chansang U, Mongkalangoon P (2019). Combined sterile insect technique and incompatible insect technique: the first proof-of-concept to suppress *Aedes aegypti* vector populations in semi-rural settings in Thailand. PLoS Negl Trop Dis.

[51] Reiter P, Nathan MB, World Health Organization, Strategy Development and Monitoring for Parasitic Diseases and Vector Control Team. Guidelines for assessing the efficacy of insecticidal space sprays for control of the dengue vector *Aedes aegypti*. World Health Organization. 2001. https://apps.who.int/iris/handle/10665/67047.

[52] Macias VM, Ohm JR, Rasgon JL (2017). Gene drive for mosquito control: where did it come from and where are we headed?. Int J Environ Res Public Health.

[53] Madon MB, Hazelrigg JE, Shaw MW, Kluh S, Mulla MS (2003). Has *Aedes albopictus* established in California?. J Am Mosq Control Assoc.

[54] Becker N, Ludwig M, Su T (2018). Lack of resistance in *Aedes vexans* (Diptera: Culicidae) field populations after 36 years of *Bacillus thuringiensis* subsp. *israelensis* (*B*.*t*.*i*) applications in the Upper Rhine Valley, Germany. J Am Mosq Control Assoc.

[55] Becker N, Jöst A, Weitzel T (2012). The *Culex pipiens* complex in Europe. J Am Mosq Control Assoc.

[56] Deutscher Wetterdienst. 2020. www.dwd.de.

[57] Deutscher Wetterdienst. 2019. www.dwd.de.

[58] Kampen H, Walther D (2017). Der Mückenatlas: Stechmücken-Monitoring mit Bürgerbeteiligung. DGaaE-Nachrichten.

[59] Gratz NG (2004). Critical review of the vector status of *Aedes albopictus*. Med Vet Entomol.

[60] Gubler DJ. Rosen L. Variation among geographic strains of *Aedes albopictus* in susceptibility to infection with dengue viruses. Am J Trop Med Hyg. 1976;25:318–25. 10.4269/ajtmh.1976.25.318 1259091

