# Correction: Integrated control of *Aedes albopictus* in Southwest Germany supported by the Sterile Insect Technique

**DOI:** 10.1186/s13071-022-05177-y

**Published:** 2022-02-18

**Authors:** Norbert Becker, Sophie Min Langentepe-Kong, Artin Tokatlian Rodriguez, Thin Thin Oo, Dirk Reichle, Renke Lühken, Jonas Schmidt-Chanasit, Peter Lüthy, Arianna Puggioli, Romeo Bellini

**Affiliations:** 1 Faculty of Biosciences, University of Heidelberg, Im Neuenheimer Feld 230, 69120 Heidelberg, Germany; 2 Institute of Dipterology (IfD), Georg-Peter-Süß-Str. 3, 67346 Speyer, Germany; 3 Kommunale Aktionsgemeinschaft zur Bekämpfung der Schnakenplage e.V. (KABS), Georg-Peter-Süß-Str. 3, 67346 Speyer, Germany; 4 IcyBac–Biologische Stechmückenbekämpfung GmbH (ICYBAC), Georg-Peter-Süß-Str. 1, 67346 Speyer, Germany; 5 Department of Arbovirology, Bernhard-Nocht-Institute for Tropical Medicine, Bernhard-Nocht-Str. 74, 20359 Hamburg, Germany; 6 Faculty of Mathematics, Informatics and Natural Sciences, Universität Hamburg, Ohnhorststrasse 18, 22609 Hamburg, Germany; 7 Institute of Microbiology, Swiss Federal Institute of Technology (ETH Zürich), Vladimir-Prelog-Weg 1-5/10, 8093 Zürich, Switzerland; 8 Centro Agricoltura Ambiente “G. Nicoli” (CAA), Via Sant’Agata 835, 40014 Crevalcore, Italy

## Correction to: Parasites & Vectors (2022) 15:9 10.1186/s13071-021-05112-7

Following publication of the original article [1], the authors flagged an error in one of the values provided in the Methods subsection ‘Quality control of the sterile *Ae. albopictus* males’: where it should say “at 25 ± 2 °C”, it said “at 252 °C”.

The published article has since been corrected.
