# Supplementary material for: Integrated control of Aedes albopictus in Southwest Germany supported by the Sterile Insect Technique
Source: Parasit Vectors. 2022 Jan 5;15:9. doi: 10.1186/s13071-021-05112-7 (PMC8727083; doi:10.1186/s13071-021-05112-7)
Supplement: Supplementary file 1 — Additional file 1: Table S1. Effect of the ratio between radiated and unirradiated males on the egg sterility rate. [file 13071_2021_5112_MOESM1_ESM.docx]

| ratio ♂ | cages | No. of | embryonated | sterile | sterility | Ø & |
| --- | --- | --- | --- | --- | --- | --- |
| fertile : sterile |  | eggs |  |  | (%) | SD |
|  | a | 93 | 92 | 1 | 1.08 |  |
| 30:0 | b | 156 | 151 | 5 | 3.21 | 5.9 |
|  | c | 82 | 71 | 11 | 13.41 | ± 6,6 |
|  | a | 45 | 26 | 19 | 42.2 |  |
| 15:15 | b | 31 | 26 | 5 | 16.12 | 30.05 |
|  | c | 132 | 90 | 42 | 31.82 | ± 13.13 |
|  | a | 97 | 12 | 85 | 87.62 |  |
| 5:25 | b | 116 | 14 | 102 | 87.93 | 85.95 |
|  | c | 79 | 14 | 65 | 82.28 | ± 3,18 |
|  | a | 60 | 11 | 49 | 81.67 |  |
| 3:30 | b | 92 | 10 | 82 | 89.13 | 84.57 |
|  | c | 117 | 20 | 97 | 82.91 | ±4.00 |
