# Supplementary material for: Integrated control of Aedes albopictus in Southwest Germany supported by the Sterile Insect Technique
Source: Parasit Vectors. 2022 Jan 5;15:9. doi: 10.1186/s13071-021-05112-7 (PMC8727083; doi:10.1186/s13071-021-05112-7)
Supplement: Supplementary file 2 — Additional file 2: Table S2. Number of Aedes albopictus eggs and percentage of sterility of the eggs in Melm (Ludwigshafen). [file 13071_2021_5112_MOESM2_ESM.docx]

| Date/ |  | **15.06.2020** | | |  | **29.06.2020** | | |  | **13.07.2020** | | |  | **27.07.2020** | | |  | **10.08.2020** | | |  | **24.08.2020** | | |  | **07.09.2020** | | |
| --- | --- | --- | --- | --- | --- | --- | --- | --- | --- | --- | --- | --- | --- | --- | --- | --- | --- | --- | --- | --- | --- | --- | --- | --- | --- | --- | --- | --- |
|  | No. of | embryo- |  |  | No. of | embryo- |  |  | No. of | embryo- |  |  | No. of | embryo- |  |  | No. of | embryo- |  |  | No. of | embryo- |  |  | No. of | embryo- |  |  |
| Trap No. | Eggs | nated | Sterile | (%) | Eggs | nated | Sterile | (%) | Eggs | nated | Sterile | (%) | Eggs | nated | Sterile | (%) | Eggs | nated | Sterile | (%) | Eggs | nated | Sterile | (%) | Eggs | nated | Sterile | (%) |
| 1A | 0 | 0 | 0 |  | 0 | 0 | 0 |  | 0 | 0 | 0 |  | 0 | 0 | 0 |  | 0 | 0 | 0 |  | 0 | 0 | 0 |  | 0 | 0 | 0 |  |
| 2A | 0 | 0 | 0 |  | 64 | 58 | 6 | 9.37% | 110 | 13 | 97 | 88.18% | 0 | 0 | 0 |  | 0 | 0 | 0 |  | 0 | 0 | 0 |  | 0 | 0 | 0 |  |
| 3A | 0 | 0 | 0 |  | 0 | 0 | 0 |  | 0 | 0 | 0 |  | 0 | 0 | 0 |  | 0 | 0 | 0 |  | 10 | 9 | 1 | 10% | 0 | 0 | 0 |  |
| 4A | 0 | 0 | 0 |  | 0 | 0 | 0 |  | 10 | 4 | 6 | 60% | 0 | 0 | 0 |  | 8 | 8 | 0 | 0% | 18 | 15 | 3 | 16.67% | 3 | 0 | 3 | 100% |
| 5A | 45 | 43 | 2 | 4.44% | 0 | 0 | 0 |  | 0 | 0 | 0 |  | 0 | 0 | 0 |  | 0 | 0 | 0 |  | 0 | 0 | 0 |  | 0 | 0 | 0 |  |
| 6A | 0 | 0 | 0 |  | 0 | 0 | 0 |  | 0 | 0 | 0 |  | 0 | 0 | 0 |  | 0 | 0 | 0 |  | 2 | 0 | 2 | 100% | 0 | 0 | 0 |  |
| 7A | 0 | 0 | 0 |  | 0 | 0 | 0 |  | 0 | 0 | 0 |  | 0 | 0 | 0 |  | 0 | 0 | 0 |  | 0 | 0 | 0 |  | 0 | 0 | 0 |  |
| 8A | 0 | 0 | 0 |  | 0 | 0 | 0 |  | 0 | 0 | 0 |  | 0 | 0 | 0 |  | 0 | 0 | 0 |  | 4 | 4 | 0 | 0% | 0 | 0 | 0 |  |
| 9A | 0 | 0 | 0 |  | 0 | 0 | 0 |  | 0 | 0 | 0 |  | 0 | 0 | 0 |  | 0 | 0 | 0 |  | 0 | 0 | 0 |  | 0 | 0 | 0 |  |
| 10A | 0 | 0 | 0 |  | 0 | 0 | 0 |  | 0 | 0 | 0 |  | 0 | 0 | 0 |  | 0 | 0 | 0 |  | 0 | 0 | 0 |  | 0 | 0 | 0 |  |
| 1B (SIT) | 0 | 0 | 0 |  | 24 | 1 | 23 | 95.83% | 46 | 13 | 33 | 71.73% | 0 | 0 | 0 |  | 0 | 0 | 0 |  | 0 | 0 | 0 |  | 45 | 0 | 45 | 100% |
| 2B (SIT) | 0 | 0 | 0 |  | 0 | 0 | 0 |  | 0 | 0 | 0 |  | 0 | 0 | 0 |  | 0 | 0 | 0 |  | 0 | 0 | 0 |  | 0 | 0 | 0 |  |
| 3B (SIT) | 0 | 0 | 0 |  | 0 | 0 | 0 |  | 0 | 0 | 0 |  | 0 | 0 | 0 |  | 7 | 5 | 2 | 28.57% | 0 | 0 | 0 |  | 0 | 0 | 0 |  |
| 4B (SIT) | 0 | 0 | 0 |  | 0 | 0 | 0 |  | 5 | 0 | 5 | 100% | 0 | 0 | 0 |  | 0 | 0 | 0 |  | 61 | 0 | 61 | 100% | 5 | 0 | 5 | 100% |
| 5B (SIT) | 0 | 0 | 0 |  | 0 | 0 | 0 |  | 0 | 0 | 0 |  | 0 | 0 | 0 |  | 0 | 0 | 0 |  | 0 | 0 | 0 |  | 0 | 0 | 0 |  |
| 6B (SIT) | 0 | 0 | 0 |  | 0 | 0 | 0 |  | 0 | 0 | 0 |  | 0 | 0 | 0 |  | 0 | 0 | 0 |  | 0 | 0 | 0 |  | 0 | 0 | 0 |  |
| 7B (SIT) | 0 | 0 | 0 |  | 0 | 0 | 0 |  | 45 | 19 | 26 | 57.77% | 0 | 0 | 0 |  | 0 | 0 | 0 |  | 15 | 0 | 15 | 100% | 40 | 0 | 40 | 100% |
| 8B (SIT) | 0 | 0 | 0 |  | 0 | 0 | 0 |  | 0 | 0 | 0 |  | 0 | 0 | 0 |  | 94 | 12 | 82 | 87.23% | 0 | 0 | 0 |  | 0 | 0 | 0 |  |
| 9B (SIT) | 0 | 0 | 0 |  | 0 | 0 | 0 |  | 0 | 0 | 0 |  | 0 | 0 | 0 |  | 0 | 0 | 0 |  | 0 | 0 | 0 |  | 0 | 0 | 0 |  |
| 10B (SIT) | 0 | 0 | 0 |  | 0 | 0 | 0 |  | 0 | 0 | 0 |  | 0 | 0 | 0 |  | 0 | 0 | 0 |  | 38 | 23 | 15 | 39.47% | 0 | 0 | 0 |  |
| 1C | 0 | 0 | 0 |  | 0 | 0 | 0 |  | 0 | 0 | 0 |  | 0 | 0 | 0 |  | 0 | 0 | 0 |  | 0 | 0 | 0 |  | 0 | 0 | 0 |  |
| 2C | 0 | 0 | 0 |  | 0 | 0 | 0 |  | 0 | 0 | 0 |  | 0 | 0 | 0 |  | 0 | 0 | 0 |  | 0 | 0 | 0 |  | 0 | 0 | 0 |  |
| 3C | 0 | 0 | 0 |  | 0 | 0 | 0 |  | 0 | 0 | 0 |  | 0 | 0 | 0 |  | 0 | 0 | 0 |  | 0 | 0 | 0 |  | 3 | 0 | 3 | 100% |
| 4C | 0 | 0 | 0 |  | 0 | 0 | 0 |  | 0 | 0 | 0 |  | 0 | 0 | 0 |  | 6 | 2 | 4 | 66.66% | 0 | 0 | 0 |  | 0 | 0 | 0 |  |
| 5C | 0 | 0 | 0 |  | 0 | 0 | 0 |  | 0 | 0 | 0 |  | 0 | 0 | 0 |  | 0 | 0 | 0 |  | 102 | 35 | 65 | 63.72% | 0 | 0 | 0 |  |
| 6C | 0 | 0 | 0 |  | 0 | 0 | 0 |  | 0 | 0 | 0 |  | 0 | 0 | 0 |  | 0 | 0 | 0 |  | 0 | 0 | 0 |  | 0 | 0 | 0 |  |
| 7C | 0 | 0 | 0 |  | 0 | 0 | 0 |  | 0 | 0 | 0 |  | 0 | 0 | 0 |  | 58 | 54 | 4 | 6.89% | 21 | 21 | 0 | 0% | 0 | 0 | 0 |  |
| 8C | 0 | 0 | 0 |  | 0 | 0 | 0 |  | 0 | 0 | 0 |  | 0 | 0 | 0 |  | 0 | 0 | 0 |  | 0 | 0 | 0 |  | 0 | 0 | 0 |  |
| 9C | 0 | 0 | 0 |  | 0 | 0 | 0 |  | 0 | 0 | 0 |  | 0 | 0 | 0 |  | 0 | 0 | 0 |  | 0 | 0 | 0 |  | 0 | 0 | 0 |  |
| 10C | 0 | 0 | 0 |  | 0 | 0 | 0 |  | 0 | 0 | 0 |  | 0 | 0 | 0 |  | 0 | 0 | 0 |  | 30 | 21 | 9 | 30% | 0 | 0 | 0 |  |
| **Total** | 45 | 43 | 2 | 4.44% | 88 | 59 | 29 | 32.95% | 216 | 49 | 167 | 77.31% | 0 | 0 | 0 | 0 | 173 | 81 | 92 | 53.18% | 301 | 128 | 171 | 56.81% | 96 | 0 | 96 | 100% |
