# Supplementary material for: Integrated control of Aedes albopictus in Southwest Germany supported by the Sterile Insect Technique
Source: Parasit Vectors. 2022 Jan 5;15:9. doi: 10.1186/s13071-021-05112-7 (PMC8727083; doi:10.1186/s13071-021-05112-7)
Supplement: Supplementary file 3 — Additional file 3: Table S3. Number of Aedes albopictus eggs and percentage of sterility of the eggs in the SIT area (Metzgergrün). [file 13071_2021_5112_MOESM3_ESM.docx]

| Date/ |  | **28.07.2020** | | |  | **11.08.2020** | | |  | **29.08.2020** | | |  | **15.09.2020** | | |  | **29.09.2020** | | |  | **13.10.2020** | | |
| --- | --- | --- | --- | --- | --- | --- | --- | --- | --- | --- | --- | --- | --- | --- | --- | --- | --- | --- | --- | --- | --- | --- | --- | --- |
|  | No. of | embryo- |  |  | No. of | embryo- |  |  | No. of | embryo- |  |  | No. of | embryo- |  |  | No. of | embryo- |  |  | No. of | embryo- |  |  |
| Trap No. | Eggs | nated | Sterile | (%) | Eggs | nated | Sterile | (%) | Eggs | nated | Sterile | (%) | Eggs | nated | Sterile | (%) | Eggs | nated | Sterile | (%) | Eggs | nated | Sterile | (%) |
| 1 | 109 | 105 | 4 | 3.7% | 21 | 1 | 20 | 95.2% | 1 | 1 | 0 | 0.0% | 2 | 0 | 2 | 100% | 0 | 0 | 0 |  | 0 | 0 | 0 |  |
| 2 | 25 | 21 | 4 | 16.0% | 60 | 48 | 12 | 20.0% | 54 | 4 | 50 | 92.6% | 9 | 9 | 0 | 0.0% | - | - | - |  | 0 | 0 | 0 |  |
| 3 | 0 | 0 | 0 |  | 0 | 0 | 0 |  | 20 | 0 | 20 | 100% | 0 | 0 | 0 |  | 0 | 0 | 0 |  | 0 | 0 | 0 |  |
| 4 | 6 | 5 | 1 | 16.7% | 63 | 22 | 41 | 65.1% | 0 | 0 | 0 |  | 0 | 0 | 0 |  | 0 | 0 | 0 |  | 0 | 0 | 0 |  |
| 5 | 0 | 0 | 0 |  | 51 | 5 | 46 | 90.2% | 75 | 46 | 29 | 38.7% | 1 | 1 | 0 | 0.0% | 0 | 0 | 0 |  | 0 | 0 | 0 |  |
| 6 | 0 | 0 | 0 |  | 0 | 0 | 0 |  | 0 | 0 | 0 |  | 1 | 1 | 0 | 0.0% | 3 | 3 | 0 | 0.0% | 4 | 0 | 4 | 100% |
| 7 | 0 | 0 | 0 |  | 44 | 21 | 23 | 52.3% | 42 | 14 | 28 | 66.7% | 23 | 1 | 22 | 95.7% | 0 | 0 | 0 |  | 0 | 0 | 0 |  |
| 8 | 70 | 37 | 33 | 47.1% | 40 | 26 | 14 | 35.0% | 86 | 8 | 78 | 90.7% | 54 | 11 | 43 | 79.6% | 0 | 0 | 0 |  | 0 | 0 | 0 |  |
| 9 | 28 | 27 | 1 | 3.6% | 31 | 27 | 4 | 12.9% | 2 | 0 | 2 | 100% | 37 | 2 | 35 | 94.6% | 1 | 1 | 0 | 0.0% | 1 | 0 | 1 | 100% |
| 10 | 12 | 12 | 0 | 0.0% | 12 | 8 | 4 | 33.3% | 22 | 7 | 15 | 68.2% | 16 | 1 | 15 | 93.8% | 9 | 9 | 0 | 0.0% | 18 | 0 | 18 | 100% |
| 11 | 20 | 20 | 0 | 0.0% | 0 | 0 | 0 |  | 13 | 11 | 2 | 15.4% | 2 | 2 | 0 | 0.0% | 0 | 0 | 0 |  | 0 | 0 | 0 |  |
| 12 | 37 | 36 | 1 | 2.7% | 12 | 0 | 12 | 100% | 14 | 11 | 3 | 21.4% | 35 | 3 | 32 | 91.4% | 21 | 14 | 7 | 33.3% | 0 | 0 | 0 |  |
| 13 | 114 | 98 | 16 | 14.0% | 0 | 0 | 0 |  | 95 | 4 | 91 | 95.8% | 17 | 15 | 2 | 11.8% | 0 | 0 | 0 |  | 0 | 0 | 0 |  |
| 14 | 127 | 112 | 15 | 11.8% | 0 | 0 | 0 |  | 1 | 0 | 1 | 100% | 14 | 0 | 14 | 100% | 4 | 0 | 4 | 100% | 0 | 0 | 0 |  |
| 15 | 15 | 8 | 7 | 46.7% | 0 | 0 | 0 |  | 24 | 0 | 24 | 100% | 77 | 7 | 70 | 90.9% | 0 | 0 | 0 |  | 0 | 0 | 0 |  |
| 16 | 0 | 0 | 0 |  | 30 | 10 | 20 | 66.7% | 0 | 0 | 0 |  | 49 | 12 | 37 | 75.5% | 47 | 4 | 43 | 91.5% | 0 | 0 | 0 |  |
| 17 | 18 | 18 | 0 | 0.0% | 4 | 0 | 4 | 100% | 29 | 2 | 27 | 93.1% | 50 | 34 | 16 | 32.0% | - | - | - | - | 0 | 0 | 0 |  |
| 18 | 41 | 30 | 11 | 26.8% | 78 | 46 | 32 | 41.0% | 71 | 7 | 64 | 90.1% | 40 | 7 | 33 | 82.5% | 44 | 24 | 20 | 45.5% | 6 | 1 | 5 | 83.3% |
| 19 | 0 | 0 | 0 |  | 23 | 12 | 11 | 47.8% | 1 | 0 | 1 | 100% | 54 | 16 | 38 | 70.4% | 0 | 0 | 0 |  | 0 | 0 | 0 |  |
| 20 | 1 | 1 | 0 | 0.0% | 0 | 0 | 0 |  | 5 | 1 | 4 | 80.0% | 0 | 0 | 0 |  | 6 | 0 | 6 | 100% | 0 | 0 | 0 |  |
| 21 | 0 | 0 | 0 |  | 6 | 4 | 2 | 33.3% | - | - | - | - | 0 | - | - | - | 0 | 0 | 0 |  | - | - | - | - |
| **Total** | 623 | 530 | 93 | 14.9% | 475 | 230 | 245 | 51.6% | 555 | 116 | 439 | 79.1% | 481 | 122 | 359 | 74.6% | 135 | 55 | 80 | 59.3% | 29 | 1 | 28 | 96.6% |
