# Supplementary material for: Integrated control of Aedes albopictus in Southwest Germany supported by the Sterile Insect Technique
Source: Parasit Vectors. 2022 Jan 5;15:9. doi: 10.1186/s13071-021-05112-7 (PMC8727083; doi:10.1186/s13071-021-05112-7)
Supplement: Supplementary file 4 — Additional file 4: Table S4. Number of Aedes albopictus eggs and percentage of sterility of the eggs in the control area (Gartenstadt). [file 13071_2021_5112_MOESM4_ESM.docx]

| Date/ |  | **29.08.2020** | | |  | **15.09.2020** | | |  | **29.09.2020** | | |  | **13.10.2020** | | |
| --- | --- | --- | --- | --- | --- | --- | --- | --- | --- | --- | --- | --- | --- | --- | --- | --- |
|  | No. of | embryo- |  |  | No. of | embryo- |  |  | No. of | embryo- |  |  | No. of | embryo- |  |  |
| Trap No. | Eggs | nated | Sterile | (%) | Eggs | nated | Sterile | (%) | Eggs | nated | Sterile | (%) | Eggs | nated | Sterile | (%) |
| 1 | 0 | 0 | 0 |  | 0 | 0 | 0 |  | 0 | 0 | 0 |  | 0 | 0 | 0 |  |
| 2 | 0 | 0 | 0 |  | 0 | 0 | 0 |  | 0 | 0 | 0 |  | 0 | 0 | 0 |  |
| 3 | 46 | 44 | 2 | 4.3% | 0 | 0 | 0 |  | - | - | - | - | - | - | - | - |
| 4 | 27 | 25 | 2 | 7.4% | 0 | 0 | 0 |  | 0 | 0 | 0 |  | 0 | 0 | 0 |  |
| 5 | 28 | 10 | 18 | 64.3% | 0 | 0 | 0 |  | 0 | 0 | 0 |  | 0 | 0 | 0 |  |
| 6 | 15 | 0 | 15 | 100% | 0 | 0 | 0 |  | 0 | 0 | 0 |  | 0 | 0 | 0 |  |
| 7 | 0 | 0 | 0 |  | 0 | 0 | 0 |  | 0 | 0 | 0 |  | 0 | 0 | 0 |  |
| 8 | 0 | 0 | 0 |  | 149 | 102 | 47 | 31.5% | - | - | - | - | - | - | - | - |
| 9 | 103 | 74 | 29 | 28.2% | 0 | 0 | 0 |  | 0 | 0 | 0 |  | 0 | 0 | 0 |  |
| 10 | 5 | 5 | 0 | 0.0% | - | - | - | - | - | - | - | - | - | - | - | - |
| 11 | 0 | 0 | 0 |  | 0 | 0 | 0 |  | 0 | 0 | 0 |  | 0 | 0 | 0 |  |
| 12 | 132 | 114 | 18 | 13.6% | 0 | 0 | 0 |  | 5 | 5 | 0 | 0.0% | 43 | 38 | 5 | 11.63% |
| 13 | 34 | 31 | 3 | 8.8% | 51 | 35 | 16 | 31.4% | 39 | 35 | 4 | 10.3% | 0 | 0 | 0 |  |
| 14 | 23 | 20 | 3 | 13.0% | 0 | 0 | 0 |  | 0 | 0 | 0 |  | 1 | 1 | 0 | 0.0% |
| 15 | 66 | 24 | 42 | 63.6% | 340 | 306 | 34 | 10.0% | 16 | 14 | 2 | 12.5% | 5 | 5 | 0 | 0.0% |
| 16 | 19 | 19 | 0 | 0.0% | 0 | 0 | 0 |  | 0 | 0 | 0 |  | 0 | 0 | 0 |  |
| 17 | 0 | 0 | 0 |  | 195 | 185 | 10 | 5.1% | 0 | 0 | 0 |  | 86 | 81 | 5 | 5.81% |
| 18 | - | - | - | - | 167 | 145 | 22 | 13.2% | 20 | 18 | 2 | 10.0% | 0 | 0 | 0 |  |
| **Total** | 498 | 366 | 132 | 26.5% | 902 | 773 | 129 | 14.3% | 80 | 72 | 8 | 10.0% | 135 | 125 | 10 | 7.41% |
